# In Vivo Evaluation of a Gastro-Resistant Enprotect^®^ Capsule under Postprandial Conditions

**DOI:** 10.3390/pharmaceutics15112576

**Published:** 2023-11-03

**Authors:** Michael Grimm, Adrian Rump, Marie-Luise Kromrey, Felix Morof, Camille Dumont, Vincent Jannin, Mladen Vassilev Tzvetkov, Werner Weitschies

**Affiliations:** 1 Department of Biopharmaceutics and Pharmaceutical Technology, University of Greifswald, 17489 Greifswald, Germany; 2 Department of Diagnostic Radiology and Neuroradiology, University Hospital Greifswald, 17475 Greifswald, Germany; 3 Department of Clinical Pharmacology, University Hospital Greifswald, 17487 Greifswald, Germany; 4 Capsugel France SAS, 68000 Colmar, France

**Keywords:** enteric hard capsule, hydroxypropyl methyl cellulose, hydroxypropyl methyl cellulose acetate succinate, MRI, caffeine, saliva, stable isotope, fed state

## Abstract

Ready-to-fill enteric hard capsule shells are an evolving field of oral drug and nutraceutical products. Lonza Capsugel^®^ Enprotect^®^ capsules were recently proven to provide reliable release in the small intestine after fasted intake, but robustness against postprandial intake needed to be proven. In this study, the capsules were administered to 16 healthy young subjects after intake of a light meal. The Enprotect^®^ capsules were labelled with 5 mg black iron oxide and 25 mg ^13^C_3_-caffeine. Magnetic Resonance Imaging was used to identify the localization and visual dispersion of the capsule filling. The salivary appearance of caffeine was considered a second independent and sensitive marker for the initial release. Whereas the fasted gastric residence time of the capsules amounted to 43 ± 32 min, it was increased to 158 ± 36 min after postprandial intake. Therefore, the mean dispersion time according to MRI and the mean caffeine appearance time were increased to 196 ± 37 min and 189 ± 37 min, respectively. But, similar to fasted administration, no capsule disintegration or leakage was observed in the stomach and 38% of the capsules disintegrated in the jejunum and 62% in the ileum. The mean dispersion time after gastric emptying and the mean caffeine appearance time after gastric emptying amounted to 38 ± 21 min and 31 ± 17 min, respectively. Both did not relevantly change compared to the fasted intake. Only the absolute dispersion time and caffeine appearance were prolonged due to the increased gastric residence and no relevant influence of the light meal was observed on the disintegration or release behavior of Enprotect^®^ capsules after gastric emptying. The capsules also showed robust enteric properties after postprandial administration.

## 1. Introduction

The oral route is a convenient and non-invasive way of administering active pharmaceutical ingredients (API) and is therefore patients’ preferred route of administration with higher compliance to medical treatments. However, many drugs undergo a loss of therapeutic activity as they pass through the acidic environment of the stomach. This is for example the case of acid-sensitive small molecules (Esomeprazole, Erythromycin, etc.) or Live Biotherapeutics Products (LBPs). Moreover, the presence of proteolytic enzymes in the gastric environment is detrimental for amino-acid-based APIs such as peptides and proteins. To ensure the therapeutic activity of these sensitive compounds, they should be administered in gastro-resistant dosage forms. These systems enable the protection of the payload from the gastric environment and the release of their contents in the distal part of the small intestine where the pH is higher and the enzymatic activity is lower than in the upper segments of the gastrointestinal (GI) tract.

Enteric dosage forms are generally obtained by depositing a layer of acid-resistant polymers on the surface of the final dosage form (e.g., tablets, capsules) or of the fill formulation (e.g., pellets). However, in addition to being a complex and sometimes costly additional step in the manufacturing of the final drug product, the conditions of the coating process can create a harsh environment for several therapeutic molecules. Indeed, it typically requires the use of heat or solvents to deposit the polymer layer, which can rapidly denature APIs such as LBP (sensitive to water) or peptides and proteins (sensitive to heat). Recently marketed Capsugel^®^ Enprotect^®^ capsules offer a smart strategy to overcome these technical constraints and facilitate the manufacturing of gastro-resistant dosage forms of sensitive therapeutic molecules. Based on hypromellose (HPMC) and HPMC Acetate Succinate (HPMC-AS), these ready-to-fill capsules formerly marketed as “Next Generation Enteric capsules” have indeed demonstrated their ability to protect a sensitive model enzyme (pancrelipase) from gastric degradation using the biorelevant Simulator of the Human Intestinal Microbial Ecosystem (SHIME, ProDigest) [1]. In addition, the enteric behavior of these capsules was confirmed in vivo in eight healthy human volunteers and showed no influence of the gastric residence time on the disintegration time after gastric emptying [2]. The results were established by two independent techniques (Magnetic Resonance Imaging (MRI) and detection of caffeine in saliva) for volunteers in a fasted state for at least 10 h before capsule intake. However, food intake leads to multiple changes in the physiological conditions of the upper part of the GI tract, which could potentially influence the behavior of Capsugel^®^ Enprotect^®^ capsules. The presence of food not only increases the gastric volume and pH, but also results in a modification of the motility pattern and a slowdown in gastric emptying [3]. Moreover, the presence of food increases the local shear rate and consequently the mechanical stress applied on enteric dosage forms.

The aim of this study was therefore to evaluate the influence of the fed state on the enteric performance of Capsugel^®^ Enprotect^®^ capsules and assess in the presence of food the location in the intestine where the capsules disintegrate. Similarly, as for evaluation in the fasted state, two independent methods—MRI observation and caffeine detection in saliva—were used to track the capsules in the GI tract of healthy volunteers. Powder containing a certain amount of ferrimagnetic black iron oxides presents different artifacts’ shape and size observable by MRI depending on their local concentration [4,5,6]. Thus, this technique enables us to distinguish the packed powder filled in the capsules from the powder dispersed in the GI tract. However, the disintegration time measured by this technique is generally overestimated as it requires the capsule to be sufficiently opened to release its content. To more precisely obtain the disintegration time of the enteric capsules, detection of caffeine in saliva was used as a second independent method. This small molecule is highly soluble in GI media and is rapidly absorbed in the small intestine, which allows caffeine detection in saliva in less than one minute after the initial release [6]. Thus, detection of caffeine in saliva correlates with the moment when the integrity of the capsules is disrupted.

## 2. Materials and Methods

### 2.1. Study Materials

Capsugel^®^ Enprotect^®^ capsules were provided by Capsugel France SAS, Colmar, France. The capsules were filled with a mixture of powders, listed in Table 1, with the following composition per capsule: 25 mg ^13^C_3_-caffeine as salivary pharmacokinetic tracer, 5 mg black iron oxide as a negative contrast agent in MRI, 35 mg croscarmellose to promote spreading of filling after disintegration and 225 mg standard capsule filling powder consisting of 99.5% mannitol and 0.5% silicon dioxide. Compared to previous studies, the amount of black iron oxide could be reduced due to the increased field strength and improved imaging parameters of the MRI. The bulk was prepared as one batch and homogenized using a bowl and pestle. Subsequently, the powder mixture was filled by hand in the capsules so as to reach a target weight of 290 mg using a capsule filling board. The capsules were tested without any post-filling treatment (no sealing, banding, nor coating).

### 2.2. Study Participants

The study was performed with sixteen healthy volunteers (nine males/seven females). All subjects gave written informed consent after they were checked for inclusion and exclusion criteria. The mean age of the volunteers was 27.4 ± 3.2 years and the mean BMI amounted to was 24.2 ± 3.4 kg/m^2^. Subjects were insured against any harm that could arise from the study procedures as well as commuting accidents. An adequate expense allowance was provided.

### 2.3. Experimental Design

The study was performed as an open-label, single-center study. Before recruitment of the subjects started, all study-related documents were checked and approved by the ethical committee at the University of Greifswald, Germany (ethical protocol No. BB 108/22). The study was registered at the German Clinical Trials Register with the code DRKS00032267.

The subjects arrived at the study unit in the morning after 10 h of fasting, 24 h of caffeine abstinence and were asked not to drink non-caloric liquids 1 h before meal intake. The time point t = 0 min is defined as the intake of the capsule. Before food intake, a blank saliva sample was withdrawn and one MR image was taken to validate an empty stomach. At t = −30 min, subjects were administered a light meal (~500 kcal) of 2 slices of a toasted sandwich, 30 g of strawberry jam, 250 g of fruit yoghurt (low fat) and 120 mL of orange juice. The subjects were instructed to finish eating breakfast in 15 min. At 14 min before the capsule intake, an additional MR image was taken and 2 min before the capsule intake, a blank saliva sample was withdrawn. At t = 0, one Enprotect^®^ capsule labelled with black iron oxide and ^13^C_3_-caffeine was administered orally in an upright position in front of the MR scanner together with 240 mL of water. Abdominal images in supine position were taken every 15 min after capsule intake. Subjects were allowed to leave the MR scanner between measurements. Saliva samples were withdrawn one minute after every imaging time point. Acquisition of the images was stopped as soon as evidence of capsule disintegration was obtained. Saliva samples were collected for 480 min. No additional food intake was allowed during the study, although still water was allowed after the capsule was located in the small intestine.

### 2.4. Salivary Sample Preparation and Evaluation of Caffeine Pharmacokinetics

The analytical method including salivary sample preparation and subsequent analysis met the criteria of the FDA Guidance for Industry “Bioanalytical Method Validation” and was performed under good laboratory practice (GLP) conditions. A detailed description of the analytical method is given in previous publications [2,7,8]. Subjects gave approximately 1 mL of saliva at each time point. All samples were frozen at −80 °C by the end of the complete sampling period after 480 min. The salivary samples were prepared by thawing and subsequent centrifugation (15 min, 18,000× *g*). Then, 100 µL of saliva was precipitated by the addition of 200 µL of 94% acetonitrile and 6% formic acid. Samples were subsequently frozen again. After thawing, these samples were again centrifuged (15 min, 18,000× *g*) before analysis. The LC-MS/MS system consisted of an Agilent 1100 series HPLC system (Agilent Technologies, Waldbronn, Germany), and a triple quadrupole mass spectrometer type API4000 QTRAP (AB Sciex, Darmstadt, Germany) using electrospray ionization source Turbo V™. The components were operated by the validated Analyst 1.6 software (AB Sciex, Darmstadt, Germany). The lower limit of quantification (LLOQ) of ^13^C_3_-labelled caffeine was 12 ng/mL. The first exceedance of this concentration of ^13^C_3_-labelled caffeine in undiluted saliva was considered as the “caffeine appearance”, from which appearance times (AT) were calculated as stated below.

### 2.5. Magnetic Resonance Imaging Sequences

**Figure 1:**
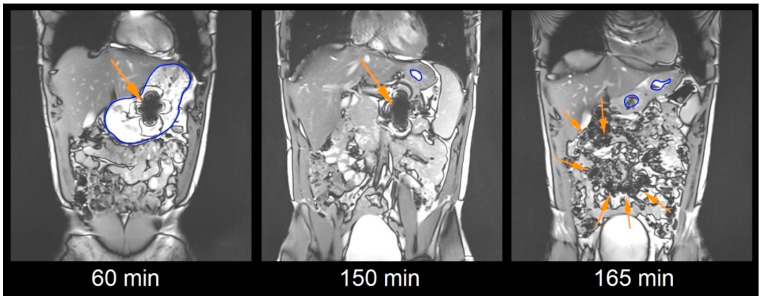
Exemplary coronal images of one volunteer with an intact capsule represented by an intact susceptibility artifact of iron oxide at 60 min after capsule ingestion in the filled stomach, after 150 min in the proximal small intestine and dispersed artifacts after disintegration of the capsule at 165 min (arrows show the respective artifact, stomach with blue demarcation).

A Siemens MAGNETOM Vida MR scanner (Siemens Healthcare, Erlangen, Germany) with a field strength of 3 Tesla was used for Magnetic Resonance Imaging. It was located in the Institute of Diagnostic Radiology and Neuroradiology. Imaging was performed with a T2 weighted TRUFI sequence with coronal slice orientation in the supine position (subject lying on the back, head forward). The repetition time was 5.6 ms, the echo time was 1.62 ms, the flip angle was 45°, slice thickness amounted to 5.0 mm with no interslice gap and the acquisition matrix was 320 × 262 scaled up to 640 × 524 with a resulting voxel size of 3.05 mm^3^. Using this TRUFI sequence, the iron oxide caused a clearly detectable susceptibility artifact as shown in Figure 1. For reduction in the motion artifacts, subjects were asked to hold their breath for up to 23 s for each image set.

### 2.6. Image Analysis

Image analysis was performed using Horos Viewer Versions 3.3.1 and 3.3.6 (The Horos Project). Each image set from a respective time point was evaluated manually for the position and integrity of the capsule by three independent and trained observers. In cases of missing concordance, these unclear findings were discussed until consensus. The integrity of the capsule shell was evaluated from the size and shape of the characteristic susceptibility artifact. The appearance of several small artifacts due to spreading or a reduction in the size or change in the geometry of the artifact was rated as dispersion of capsule filling. For dispersion to occur, the capsule needed to be disintegrated to a relevant extent. Therefore, dispersion of filling is regarded a good estimate for capsule disintegration [2]. Exemplary images of subject 13 are given in Figure 1.

### 2.7. Capsule Evaluation Criteria

**Figure 2:**
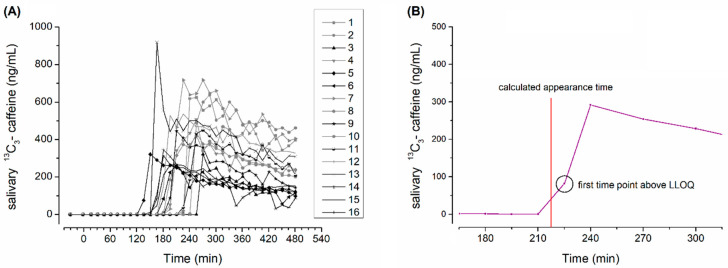
(**A**): Individual salivary caffeine concentrations with male subjects in black and females in grey (n = 16); (**B**): amplified exemplary caffeine profile with marking of calculated appearance time.

The filling dispersion time (DT), the gastric residence time (GRT) and site of dispersion were evaluated by MRI. The salivary ^13^C_3_-caffeine appearance time (AT) was assessed from salivary pharmacokinetics and exceedance of LLOQ. The DT as seen in MRI and salivary caffeine AT of the capsules were determined as the mean between the first measurement time point dispersion or caffeine appearance and the last time point before this observation. An example of evaluation of AT is given in Figure 2.

The gastric residence time (GRT) was also defined as the mean of the last time point the capsule was still located in the stomach and the first time point the capsule was not located in the stomach anymore. The site of dispersion was determined as the region of the gastrointestinal tract in which the capsule was located at the time of the detected dispersion [2].

The prolongation of gastric residence was likely to also prolong disintegration, if the capsules resist postprandial conditions in the stomach. Moreover, the subsequent erosion after gastric emptying might be affected after longer gastric residence with an increased pH. Thus, the dispersion time after gastric emptying (DT after GE) and the appearance time after gastric emptying (AT after GE) were calculated by subtracting the GRT from the DT or AT, respectively. These parameters were formerly referred to as the Time to Disintegration after GE [6,9] or intestinal transit time of the capsules until disintegration (ITT_D) [2].

### 2.8. Statistical Analysis

Dispersion times (DT), caffeine appearance times (AT) and gastric residence times (GRT) are given as individual data in the graphs and as mean ± standard deviation in continuous text. The statistical evaluations were performed using Excel 2019, GraphPad Prism 5 and OriginPro 8.5.1. Gaussian distribution was tested by the Shapiro–Wilk normality test. To evaluate the effect of gastric residence on the subsequent disintegration rate, a correlation test of the GRT and DT after GE as well as a correlation test of GRT and AT after GE were performed. Since no Gaussian distribution could be assured and a non-linear relationship seemed possible, the Spearman correlation test was performed in addition to the common linear Pearson correlation test. The comparison of DT after GE and AT after GE was performed by a paired non-parametric Wilcoxon signed rank test. The comparison of DT after GE following the fasted and postprandial intake of the capsules was performed by an unpaired non-parametric Mann–Whitney test. Differences were accepted as significant if *p* < 0.05.

## 3. Results and Discussion

All subjects successfully completed the study and no subject experienced any adverse effects related to the study procedures. All saliva samples and all images from MRI could be evaluated. In some cases, we had technical problems with abdominal coils, but the images were still evaluable. The individual salivary profiles are depicted below in Figure 2.

**Figure 3:**
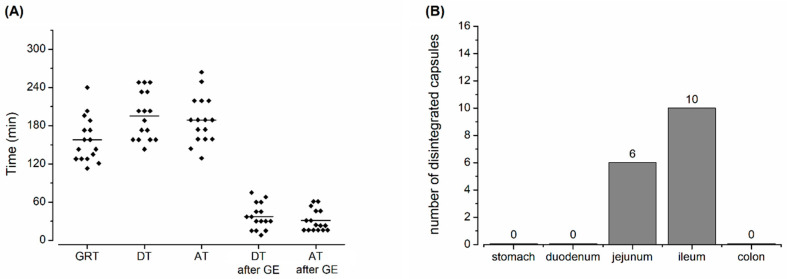
(**A**) Dispersion time (DT) as determined by MRI and ^13^C_3_-labelled caffeine appearance time (AT) in saliva, with gastric residence times (GRT) and DT and AT relative to gastric emptying, mean times marked by a horizontal bar (n = 16 each). (**B**) The site of dispersion as obtained from MRI tracking.

From salivary pharmacokinetics, the ^13^C_3_-caffeine appearance times (AT) were obtained. Together with the respective GRT and the DT as evaluated by MRI, the ATs are shown in Figure 3. Moreover, the DT after GE and AT after GE are given. No sign of disintegration in the stomach was observed for any capsule. According to the evaluation of the MRI data sets, the disintegration of the capsules took place in the jejunum in six subjects and in the ileum in ten subjects. After fasted intake, three of eight capsules disintegrated in the jejunum and five of eight in the ileum or colon [2], which are exactly the same percentages reported for the fed state. Absolute numbers doubled in the fed state due to the doubled sample size. Colonic disintegration after fasted intake could be considered as an extreme case as it was only attributed to extremely fast intestinal transit and not to prolonged disintegration of the capsule. Thus, it can be concluded that meal intake did not influence the site of disintegration.

The specific intestinal region of delivery could be of special interest for certain drugs as regional differences between the intestinal compartments exist. The different expression of transporters and enzymes could play a role for specific drug substances delivered by such a capsule [10]. Moreover, the pH gradually rises from the duodenum to the jejunum to the ileum. This in turn is also the factor that promotes the dissolving of the enteric polymers used for manufacturing, but also plays a role in the ionization of a potential drug, thus affecting its solubility and absorption. Also, the effective surface area for absorption differs. For example, the ileal surface area enlargement is approximately only half of that in the jejunum [11]. Nonetheless, slower absorption related to a slightly smaller surface area might be partially compensated by the longer length and residence times in the ileum. Moreover, there is no sharp delineation between the jejunum and ileum and rapid transport to deeper segments after disintegration occurs in the small intestine. Also, retrograde mixing motility can move chyme and liquids in which a drug could be dispersed from the ileum back to the jejunum. Thus, disintegration in the distal jejunum or proximal ileum is probably without practically relevant differences even for drugs with more limited absorption than our model drug caffeine. In this regard, the Enprotect^®^ capsules show reproducible delivery to an intestinal region. The individual raw data on salivary caffeine can be found as a supplementary file.

The mean DT according to MRI amounted to 196 ± 37 min. With 189 ± 37 min, the caffeine AT was only slightly earlier, indicating that the salivary caffeine method is more sensitive for the initial release and small leakages that do not necessarily lead to complete dispersion of filling. This is in line with previous findings [2,6]. Moreover, this difference is the reason to specifically call the visualized process in MRI “dispersion”, and the exceedance of ^13^C_3_-caffeine in saliva “appearance”, since both processes only indicate a step of disintegration, but not disintegration itself. Both detection methods provided very comparable results and confirmed a robust enteric formulation even in the fed state. Other labelling methods like tracking of oil-filled capsules or the utilization of perfluorated substances could provide other views on the disintegration of gastro-resistant dosage forms but require other specific sequences, equipment and experience [12,13]. Individual MR images from our study for complete tracking of the capsules labelled with black iron oxide can be found as supplementary files.

In contrast to the fasted GRT, which only amounted to 43 ± 32 min, the postprandial GRT of the capsules was increased to 158 ± 36 min. Surprisingly, this is shorter than the mean GRT of 210 ± 84 min of enteric capsules from a previous study with a similar meal [6]. Nonetheless, this GRT is within the estimated range. Such monolithic objects are mainly emptied from the stomach by the housekeeping waves of the interdigestive migrating motor complex (IMMC). This motility pattern is only present in the fasted state. Thus, for the emptying of the capsules, the subjects need to fast again. The gastric emptying rate of a meal is calorie dependent and according to the literature, the emptying rate amounts to 2–4 kcal/min [14,15]. With the respective meal of approx. 500 kcal used in this study, it would take about 125 min to 250 min to re-achieve the fasted state, and the possible recurrence of motility patterns able to empty the capsule. Interestingly, with a range of GRT between 113 and 240 min, gastric emptying of the capsules exactly occurred within this timespan.

Related to the calorie amount of an administered meal, the gastric emptying time of such a monolithic dosage form is very predictable. Due to the reproducible subsequent disintegration behavior in small intestines, the start of absorption could also be timed well using the Capsugel^®^ Enprotect^®^ capsule.

The long gastric residence led to a pronounced prolongation of disintegration, but to obtain better insight into the subsequent release and disintegration processes after gastric emptying, the GRT was subtracted from the DT and AT, so that only the small intestinal transit time until disintegration can be evaluated. The mean DT after GE amounted to 38 ± 21 min and the mean AT after GE was again shorter with 31 ± 17 min. A Wilcoxon signed rank test revealed no significant difference (*p* = 0.20). The disintegration behavior of the Capsugel^®^ Enprotect^®^ capsules might also be faster and more homogenous than that of common coated tablets. According to the literature, exemplary enteric Naproxen tablets showed a DT after GE of 94 ± 36 min (n = 14) after the intake of a light meal as evaluated by scintigraphy [9]. Nonetheless, there might be faster enteric tablet formulations available with more homogenous disintegration, but comparable in vivo data are scarce. Nevertheless, these enteric capsules are a ready-to-use solution, with no necessity to develop a specific formulation or coating process.

Typically, one would assume an increased risk of dose dumping due to prolonged gastric residence, increased pH and more mechanical stress. Accelerated disintegration after postprandial gastric residence of such a dosage form also seems likely.

Telemetric capsule data in human stomachs show that even with a larger meal with more fat and proteins, the capsule is surrounded by media with pH 4 or less already a few minutes after intake. Moreover, on average 1.1 ± 1.1 h after intake, such a capsule can already face media with pH 2 and below [16]. Thus, the pH even after higher caloric meals does not seem to be high enough to provoke sufficient dissolution of the enteric polymers, causing the shell to break. On the other side, the presence of digested food has more implications on the surrounding media of a dosage form besides pH that could increase the erosion rate of the capsule shell. Due to local pH effects around the dosage form, an increased buffer capacity could improve the dissolution of acidic gastro-resistant polymers even at lower pHs, as long as it is slightly above the polymers’ pKa. But nonetheless, since the pKa of HPMC-AS used in the evaluated capsules is approximately 5, the effects of the media’s pH and buffer capacity on the erosion of Enprotect^®^ capsules are probably limited [17]. Besides the effects of increased pH, buffer capacity and mechanical stress, other factors could increase the speed of the disintegration and dissolution of oral dosage forms in the fed state too [18]. Due to increased postprandial bile salt contents and lipolysis products, the surface tension of the postprandial media is lower, which in turn could favor the wetting of the capsule’s surface, penetration into small cracks and an increase in dissolution rates [19,20,21]. On the other side, increased postprandial viscosity might slow down the dissolving of the enteric polymer shell.

Since the capsule is only emptied after the reappearance of fasted state motility (MMC phase 3), it will most likely enter an empty upper small intestine. Imaging data suggest that it is very unlikely that capsules face chyme until they arrive in the ileum. This is supported by results from telemetric capsule and scintigraphic studies showing similar intestinal pH profiles or transit behavior after fasted and postprandial administration [22,23]. The composition of intestinal media available for erosion and disintegration of monolithic gastro-resistant dosage forms like the Enprotect^®^ capsules is therefore most likely to be comparable to the fasted state for most of the transit. Thus, altered parameters of media composition in the small intestine should not be overestimated for the dissolution of enteric dosage forms as long as they withstand the gastric conditions.

**Figure 4:**
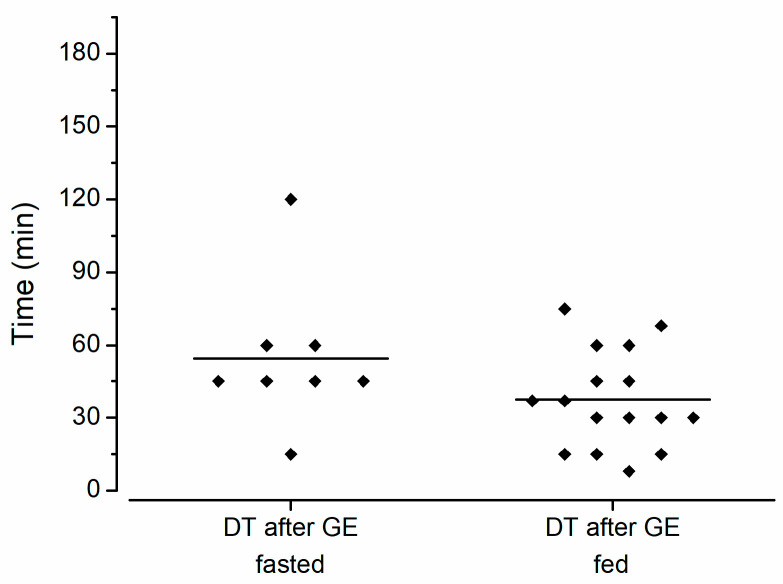
Dispersion time after gastric emptying (DT after GE) as determined by MRI after intake of Enprotect^®^ capsules in fasted (n = 8) and fed state (n = 16). The mean disintegration time is marked by a horizontal bar. Fasted data from previous publication [2].

In addition, the DT after GE was not relevantly affected by meal intake, suggesting no pronounced influence of prolonged gastric residence or intestinal media composition on subsequent disintegration behavior. As can be seen in Figure 4, the DT after GE after the fasted intake amounted to 54 ± 30 min, whereas the DT after GE following postprandial intake amounted to 38 ± 20 min. Although the DT after GE in the fed state is slightly shorter, an unpaired non-parametric Mann–Whitney test revealed no significant difference (*p* = 0.14). It must be taken into account that only a light meal was used in this study. With a heavier meal, GRT, the pH and mechanical stress might be increased to a larger extent [16]. It is unclear whether the capsule could also withstand these conditions.

**Figure 5:**
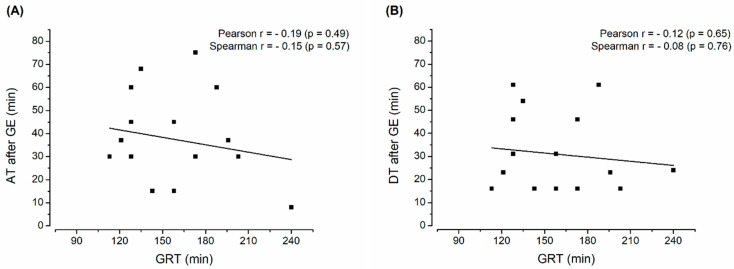
(**A**) Dispersion time after gastric emptying (DT after GE) and (**B**) caffeine appearance time after gastric emptying (AT after GE). Correlation is marked by a line; correlation coefficients and corresponding *p*-values are stated. (n = 16).

Nonetheless, the capsule did not show any leakages even during longer gastric residence of up to 240 min and no influence of prolonged gastric residence on subsequent disintegration or release behavior. This missing correlation of the GRT and DT after GE as well as the AT after GE is shown in Figure 5. The missing correlation indicates that even longer GRTs are not likely to cause the failure of this enteric capsule system.

## 4. Conclusions

In this study, the in vivo performance of Lonza Capsugel^®^ Enprotect^®^ capsules was evaluated in sixteen healthy subjects after intake in the postprandial state. MRI and salivary ^13^C-caffeine appearance were used as two independent and complementing methods. Even under fed conditions, the Capsugel^®^ Enprotect^®^ capsules showed robust gastro-resistant and enteric disintegration properties by use of both methods independently.

Although administration in the fasted state might be favorable, an intended or unintended postprandial intake of the capsules would not cause the failure of the gastro-resistant system.

## Figures and Tables

**Table 1:** Ingredients of the investigated capsules like in the previous study [2].

Ingredient	Producer/Distributor
Capsugel^®^ Enprotect^®^ capsules size 0	Capsugel France SAS, Lonza group, Colmar, France
Black iron oxide E172	Caesar & Loretz GmbH, Hilden, Germany
^13^C_3_-labelled caffeine	SIGMA-ALDRICH CHEMIE GmbH, Schnelldorf, Germany
Croscarmellose, Ph.Eur.	JRS Pharma GmbH & Co. KG, Rosenberg, Germany
Mannitol, Ph.Eur.	Fagron GmbH & Co. KG, Barsbüttel, Germany
Silicon dioxide, Ph.Eur.	Fagron GmbH & Co. KG, Barsbüttel, Germany

## Data Availability

The data presented in this study are available in respective supplementary materials of this publication.

## Supplementary Materials

The following supporting information can be downloaded at: https://www.mdpi.com/article/10.3390/pharmaceutics15112576/s1, Files 1–17: MRI data subject 001, MRI data subject 002, MRI data subject 003, MRI data subject 004, MRI data subject 005, MRI data subject 006, MRI data subject 007, MRI data subject 008, MRI data subject 009, MRI data subject 010, MRI data subject 011, MRI data subject 012, MRI data subject 013, MRI data subject 014, MRI data subject 015, MRI data subject 016, individual salivary caffeine concentrations
