# Supplementary material for: In Vivo Evaluation of a Gastro-Resistant Enprotect® Capsule under Postprandial Conditions
Source: Pharmaceutics. 2023 Nov 3;15(11):2576. doi: 10.3390/pharmaceutics15112576 (PMC10674880; doi:10.3390/pharmaceutics15112576)
Supplement: Supplementary file 1 [file pharmaceutics-15-02576-s001.zip › pharmaceutics-2602663 - supplementary file17.pdf]

Salivary caffeine concentrations

Time (min)	Salivary caffeine concentration (ng/mL)							
	subject 001	subject 002	subject 003	subject 004	subject 005	subject 006	subject 007	subject 008
-31	0	0	0	0	0	0	0	0
-2	0	0	0	0	0	0	0	0
16	0	0	0	0	0	0	0	0
31	0	0	0	0	0	0	0	0
46	0	0	0	0	0	0	0	0
61	0	0	0	0	0	0	0	0
76	0	0	0	0	0	0	0	0
91	0	0	0	0	0	0	0	0
106	0	0	0	0	0	0	0	0
121	0	0	0	0	0	0	0	0
136	0	0	0	0	75	0	0	0
151	0	0	0	0	321	0	0	0
166	0	0	0	0	291	0	0	0
181	0	10	0	0	262	84	0	0
196	0	185	0	53	262	224	29	0
211	0	315	0	342	250	260	465	0
226	5	372	0	393	224	217	717	0
241	618	357	0	435	209	144	639	3
256	624	435	0	507	178	206	645	489
271	555	375	321	342	183	226	717	600
286	579	375	233	375	164	185	648	639
301	612	351	246	324	162	163	660	504
316	564	339	194	324	164	152	567	525
331	450	309	198	234	144	185	510	600
346	414	318	176	327	152	142	555	558
361	432	291	202	296	141	168	483	477
376	495	280	165	284	137	147	432	492
391	429	274	104	296	137	152	471	480
406	342	265	116	268	139	145	534	501
421	378	258	114	266	143	127	471	492
436	420	243	122	234	127	117	402	453
451	342	250	136	220	129	56	429	462
466	366	233	134	261	117	93	393	438
481	396	238	123	235	97	113	405	462

Time (min)	Salivary caffeine concentration (ng/mL)							
	subject 009	subject 010	subject 011	subject 012	subject 013	subject 014	subject 015	subject 016
-31	0	0	0	0	0	0	0	0
-2	0	0	0	0	0	0	0	0
16	0	0	0	0	0	0	0	0
31	0	0	0	0	0	0	0	0
46	0	0	0	0	0	0	0	0
61	0	0	0	0	0	0	0	0
76	0	0	0	0	0	0	0	0
91	0	0	0	0	0	0	0	0
106	0	0	0	0	0	0	0	0
121	0	0	0	0	0	0	0	0
136	0	0	0	0	0	0	0	0
151	0	0	0	0	0	0	0	13
166	0	0	0	0	918	86	111	47
181	0	0	0	152	555	345	315	209
196	88	0	0	528	441	309	266	264
211	444	0	0	468	510	266	264	251
226	408	19	37	537	447	222	262	251
241	360	372	186	498	501	227	237	228
256	369	420	432	468	501	190	231	224
271	357	339	450	468	474	149	245	202
286	275	390	420	450	471	172	216	184
301	284	363	378	465	417	174	188	193
316	281	468	351	384	450	199	185	172
331	261	453	375	450	423	146	170	170
346	231	306	330	408	414	45	158	155
361	218	249	321	423	321	90	169	133
376	233	271	312	381	354	111	149	138
391	209	265	294	375	339	137	156	131
406	159	264	372	360	289	130	132	124
421	227	257	306	348	342	137	146	138
436	190	236	272	333	299	32	146	128
451	168	209	245	339	278	50	151	143
466	152	209	272	336	312	37	154	119
481	143	201	209	327	309	87	152	122
