# Supplementary figures and images for: In Vivo Evaluation of a Gastro-Resistant Enprotect® Capsule under Postprandial Conditions
Source: Pharmaceutics. 2023 Nov 3;15(11):2576. doi: 10.3390/pharmaceutics15112576 (PMC10674880; doi:10.3390/pharmaceutics15112576)

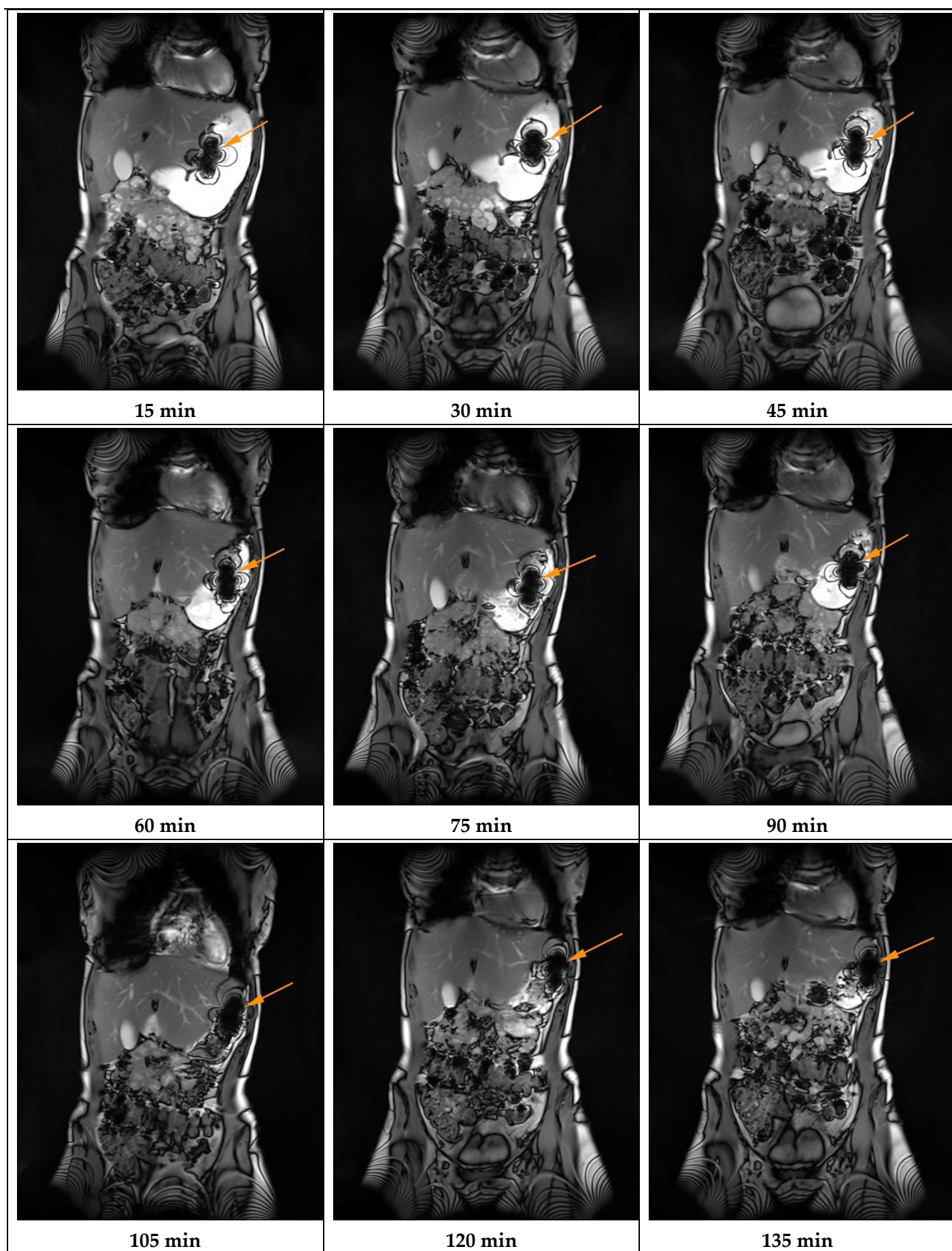

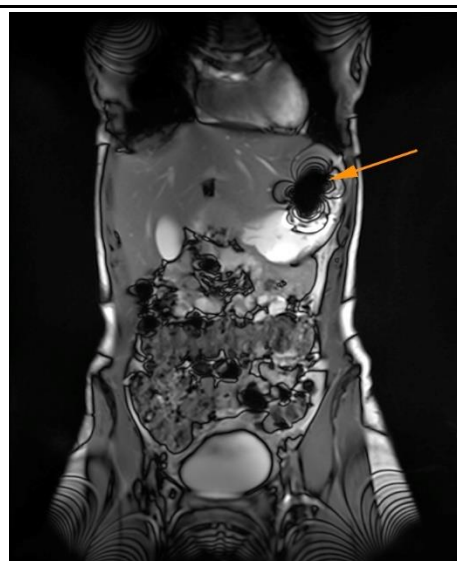

150 min

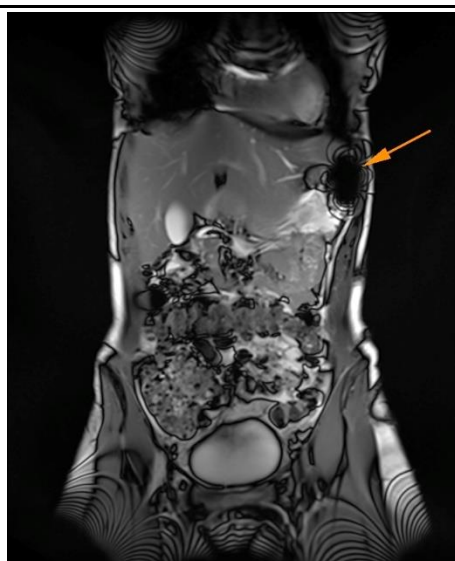

165 min

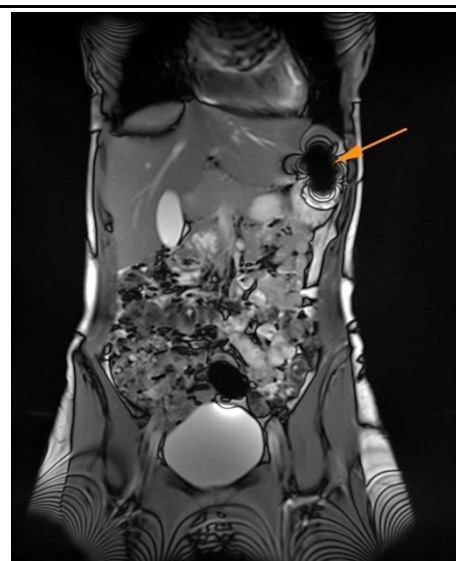

180 min

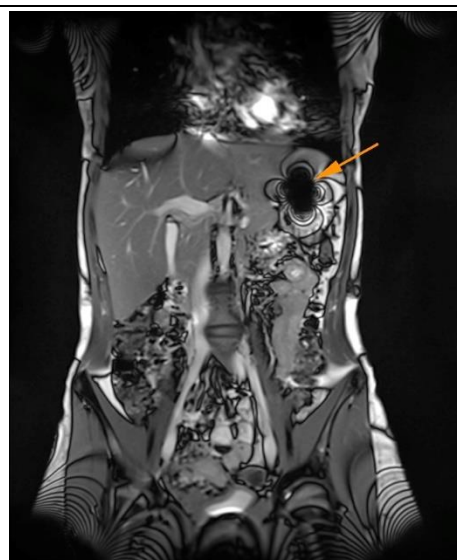

195 min

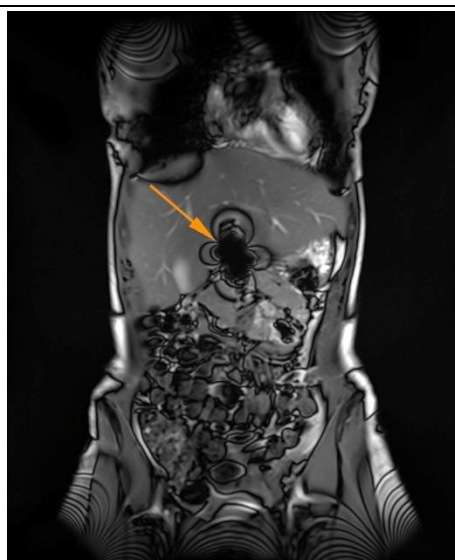

210 min

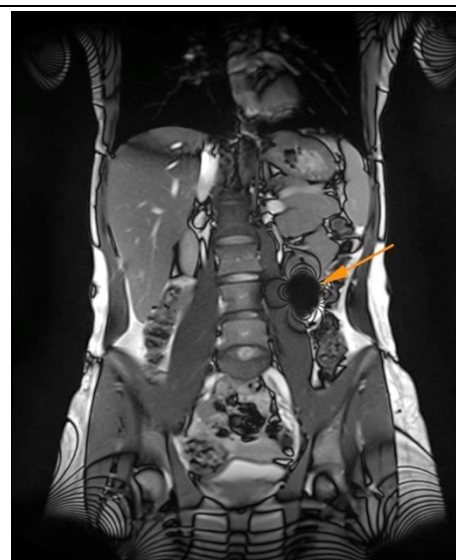

225 min

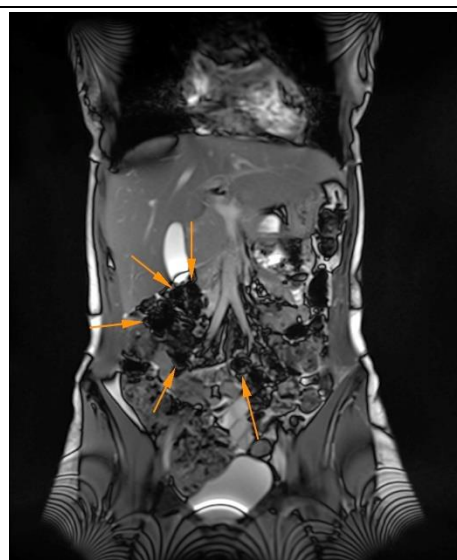

240 min

Supplement: Supplementary file 1 [file pharmaceutics-15-02576-s001.zip › pharmaceutics-2602663 - supplementary file1.pdf]

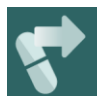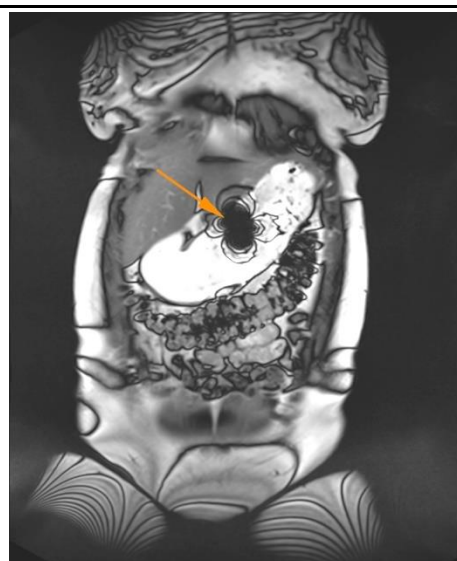

15 min

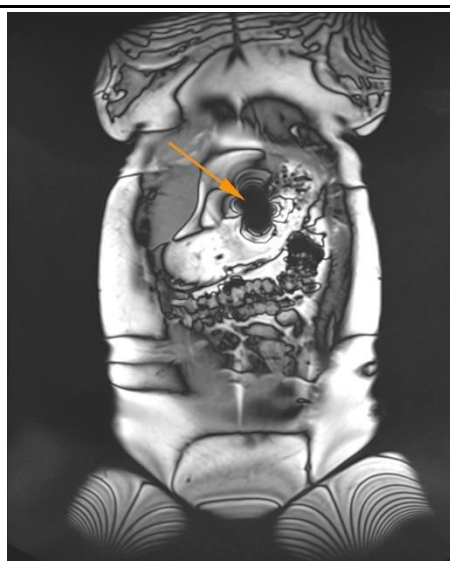

30 min

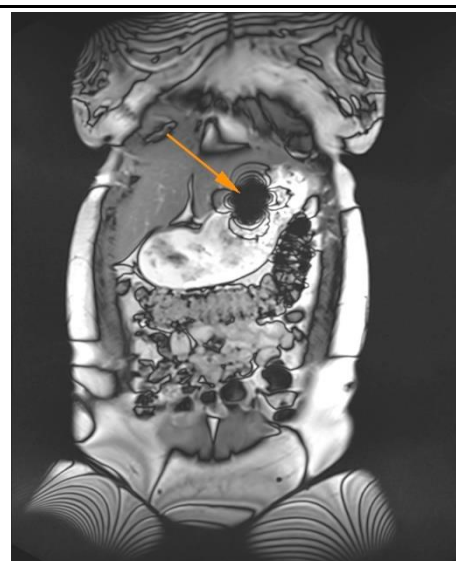

45 min

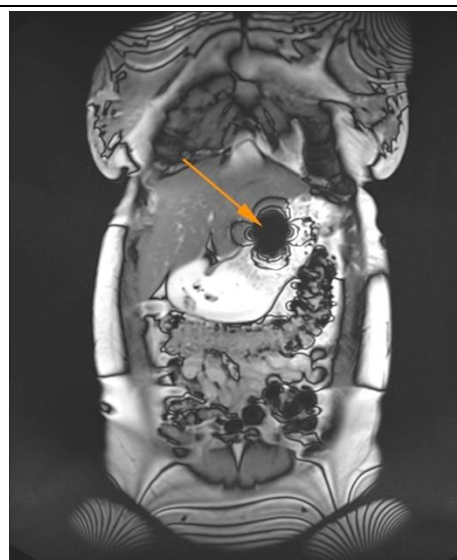

60 min

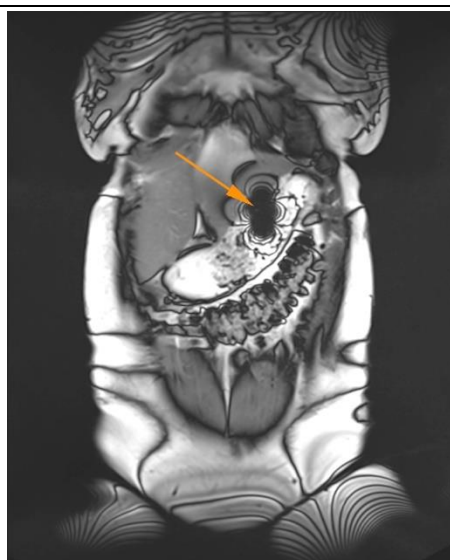

75 min

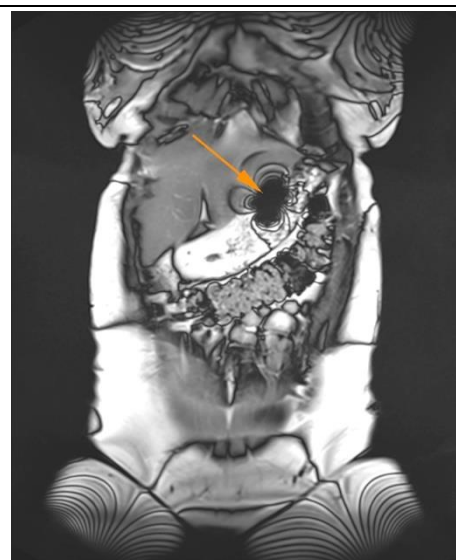

90 min

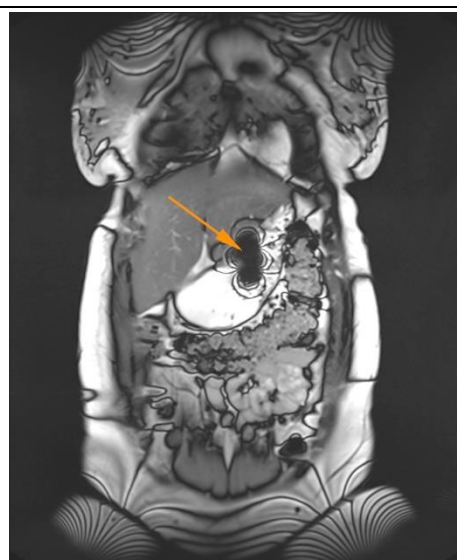

105 min

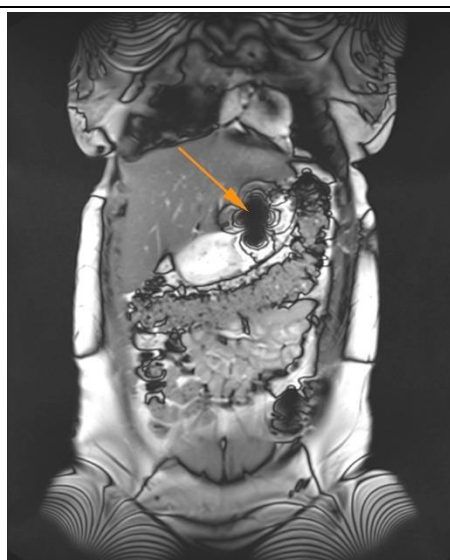

120 min

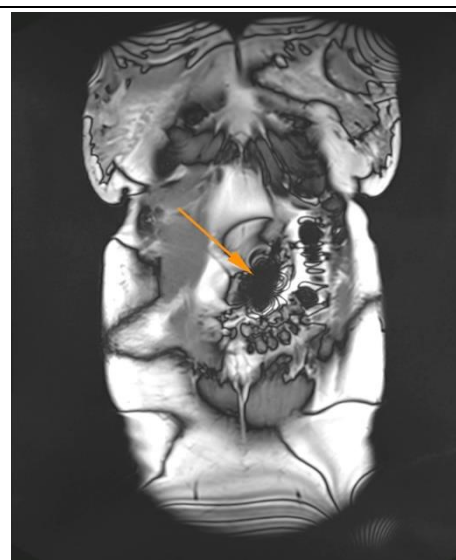

135 min

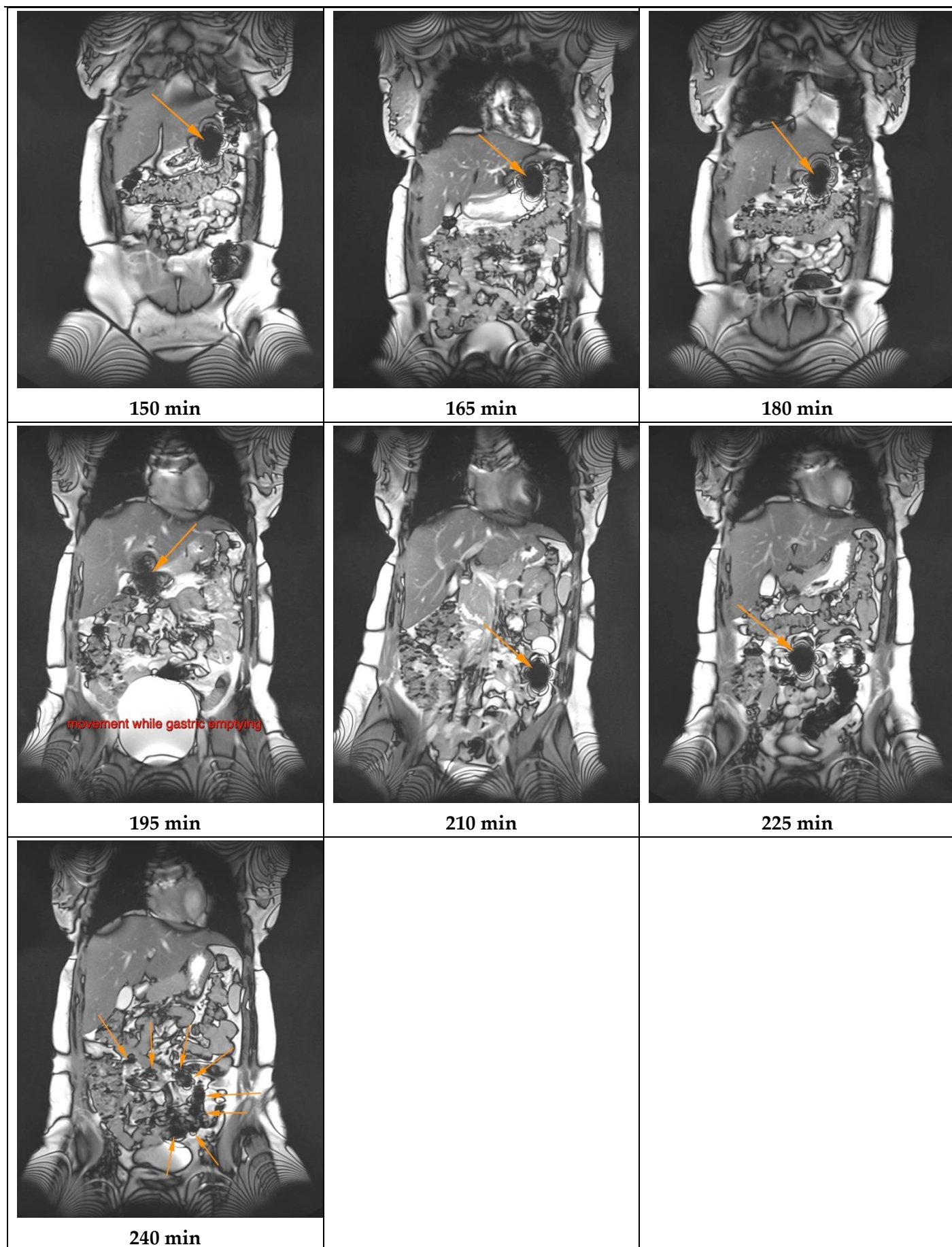

Supplement: Supplementary file 1 [file pharmaceutics-15-02576-s001.zip › pharmaceutics-2602663 - supplementary file10.pdf]

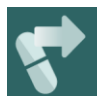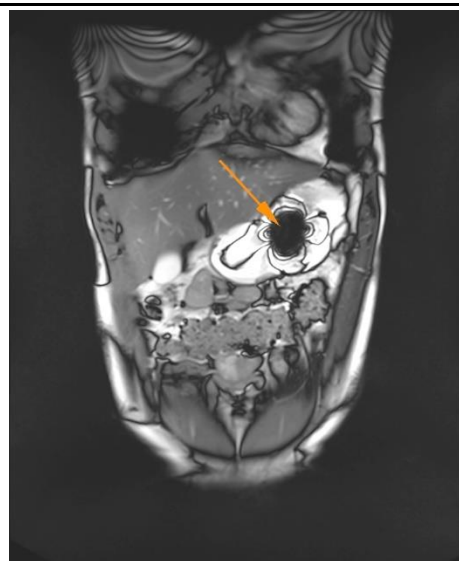

15 min

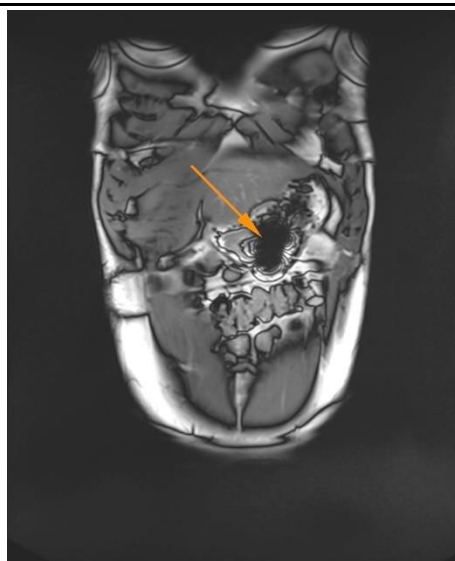

30 min

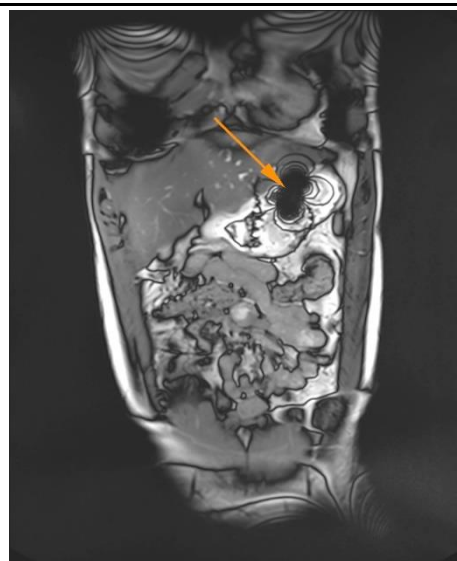

45 min

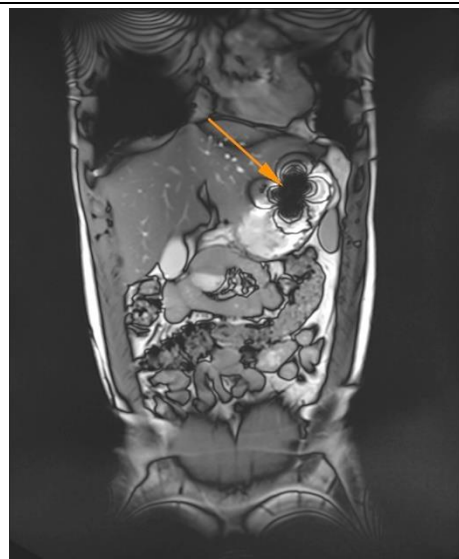

60 min

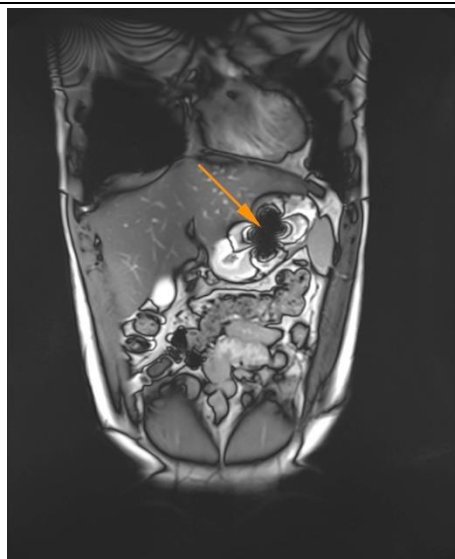

75 min

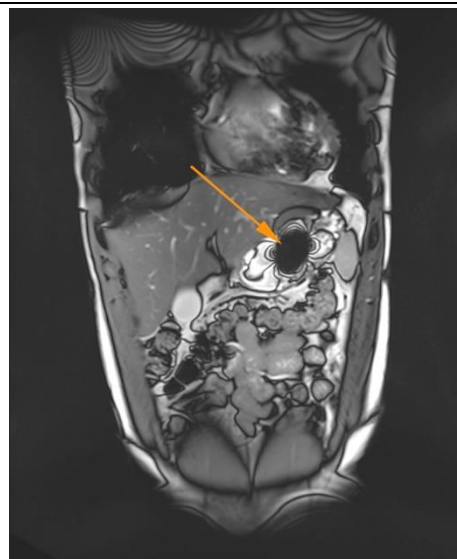

90 min

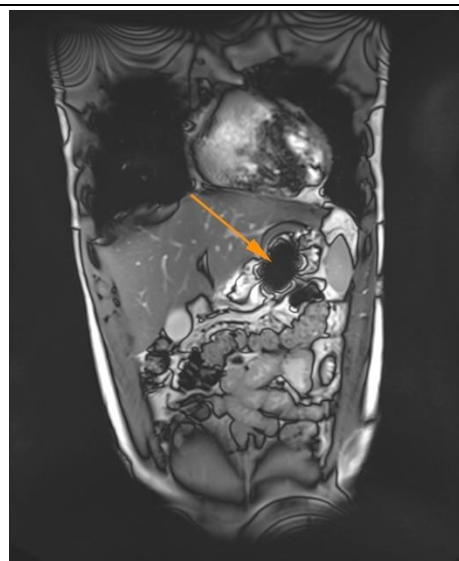

105 min

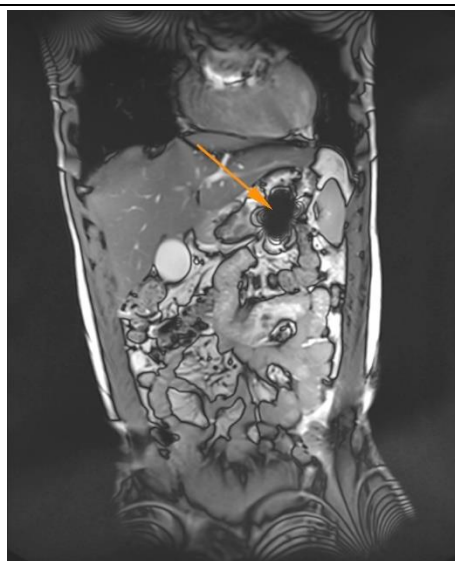

120 min

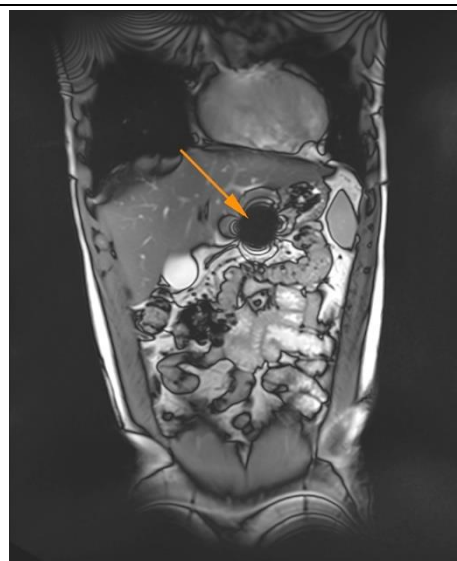

135 min

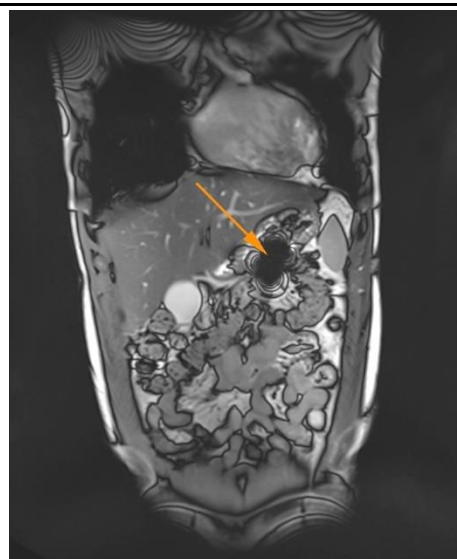

150 min

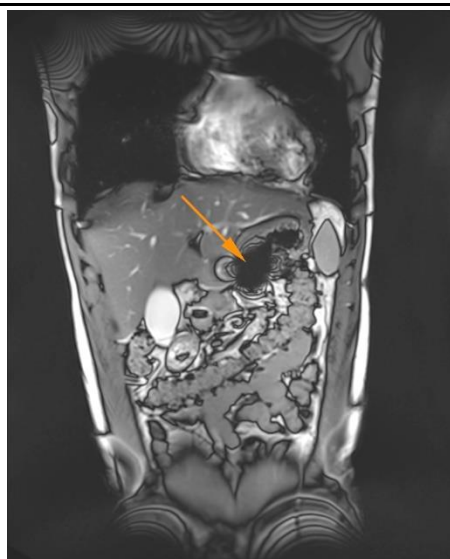

165 min

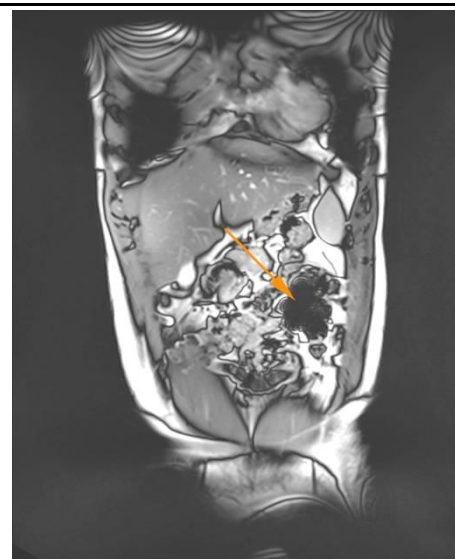

180 min

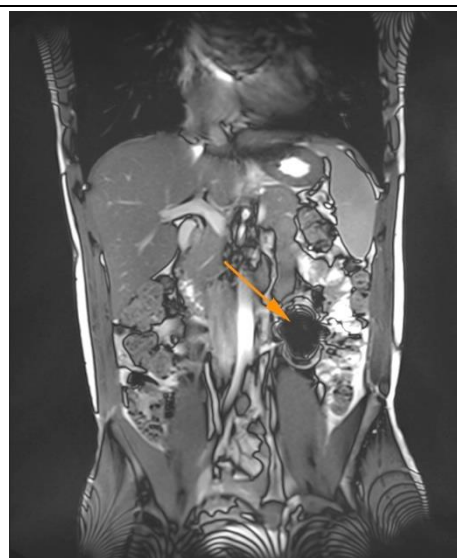

195 min

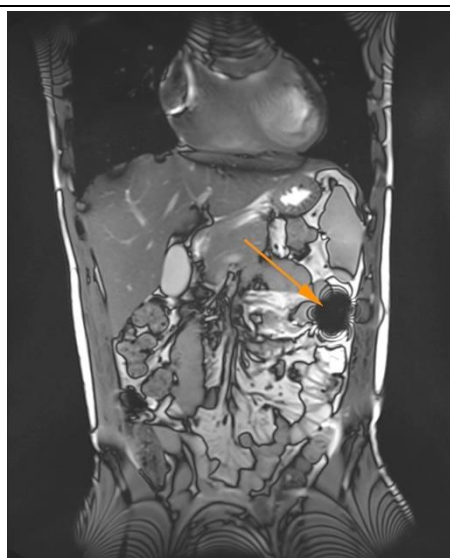

210 min

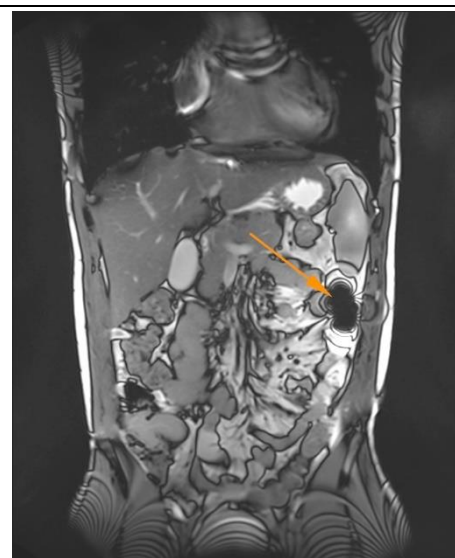

225 min

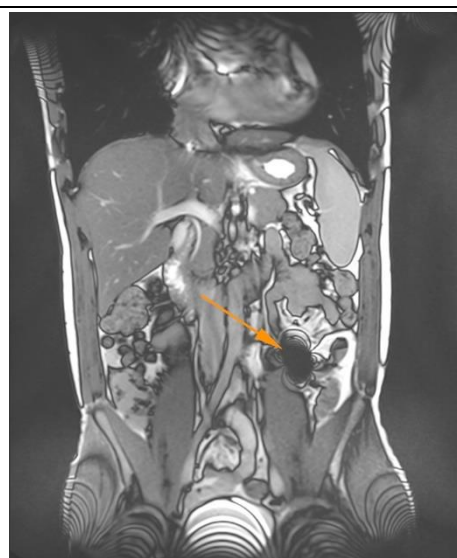

240 min

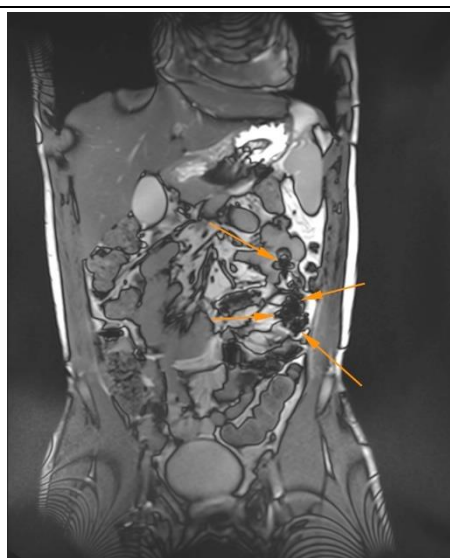

255 min

Supplement: Supplementary file 1 [file pharmaceutics-15-02576-s001.zip › pharmaceutics-2602663 - supplementary file11.pdf]

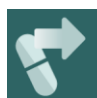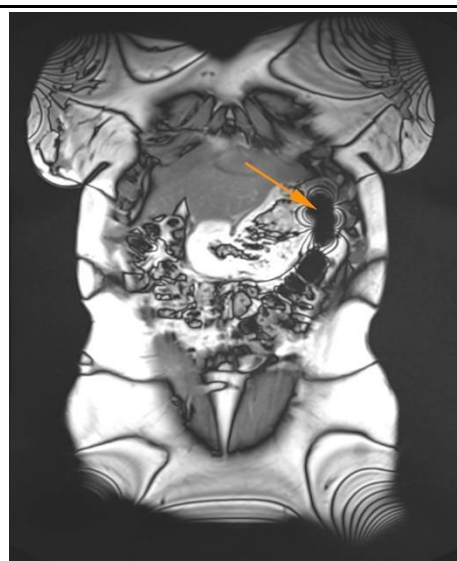

15 min

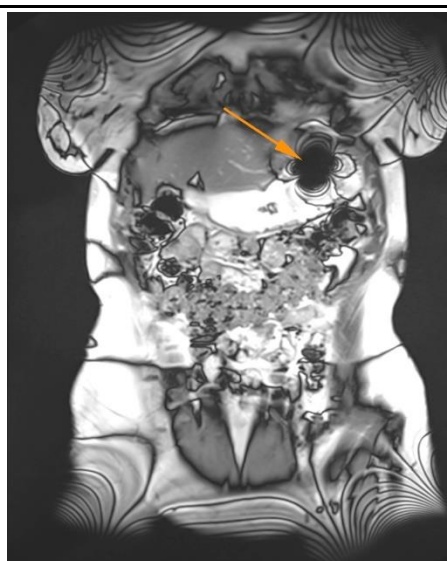

30 min

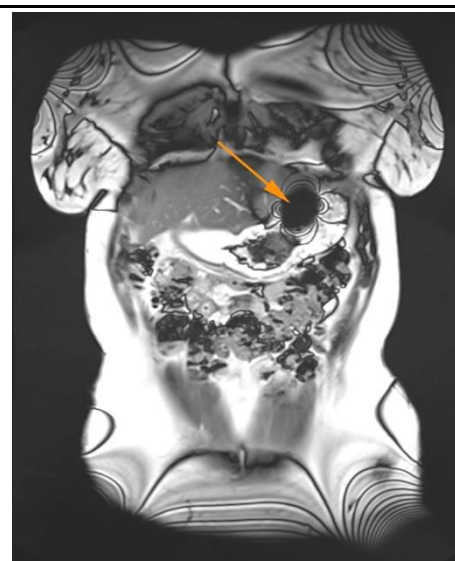

45 min

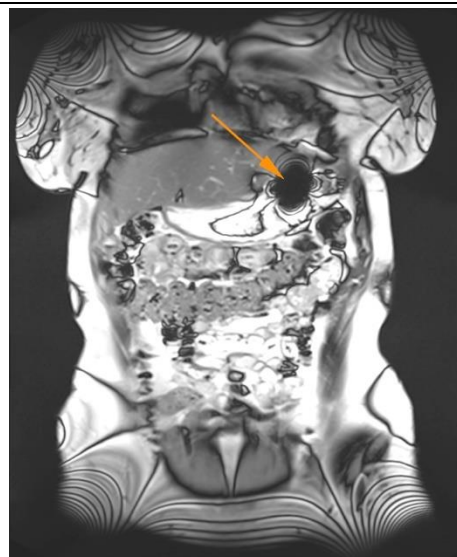

60 min

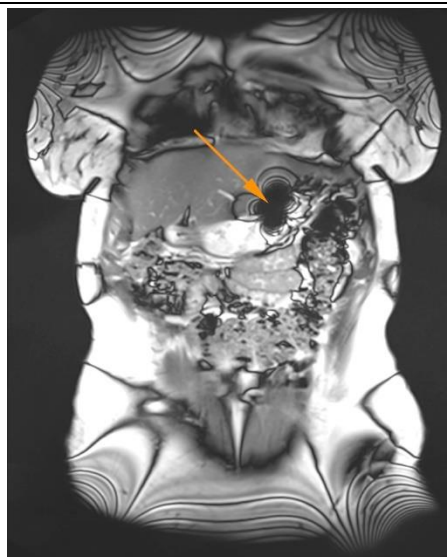

75 min

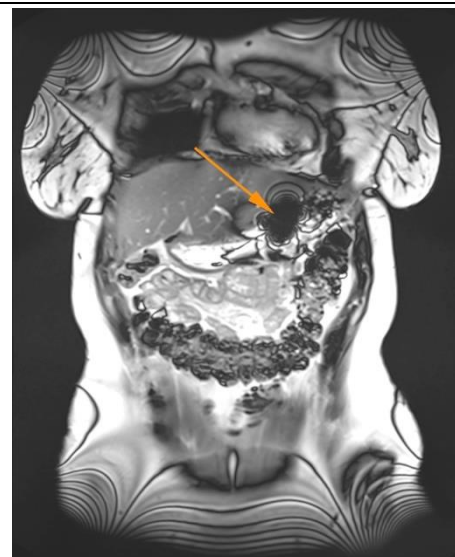

90 min

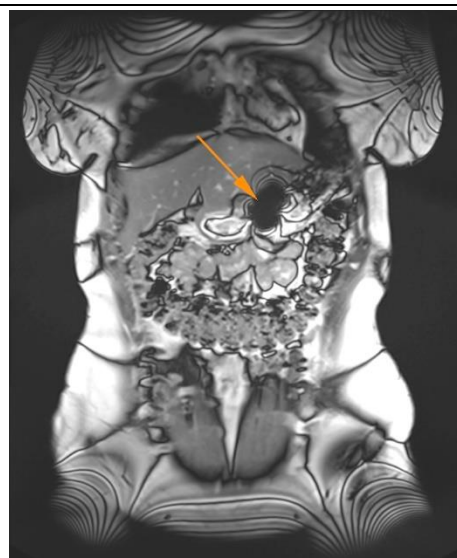

105 min

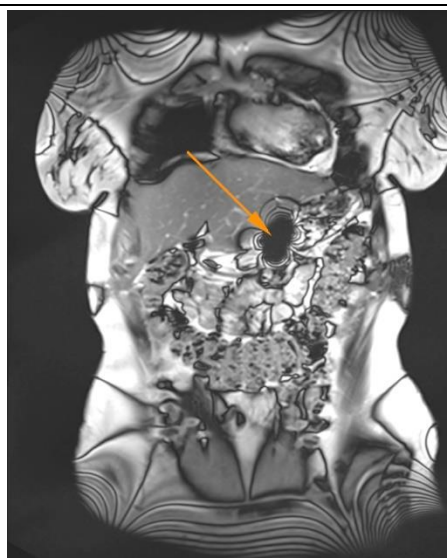

120 min

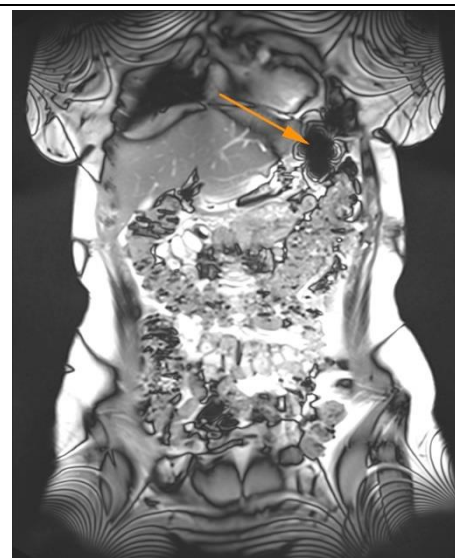

135 min

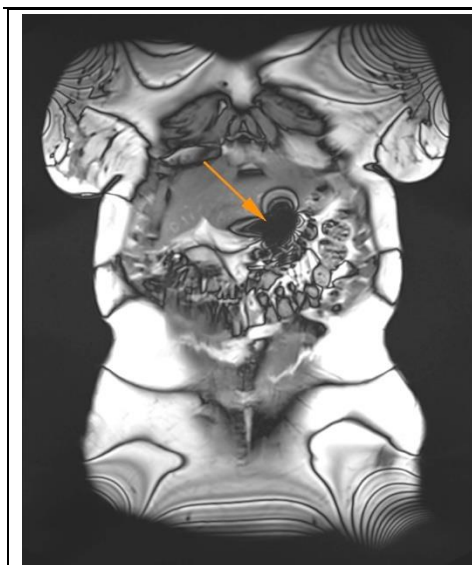

150 min

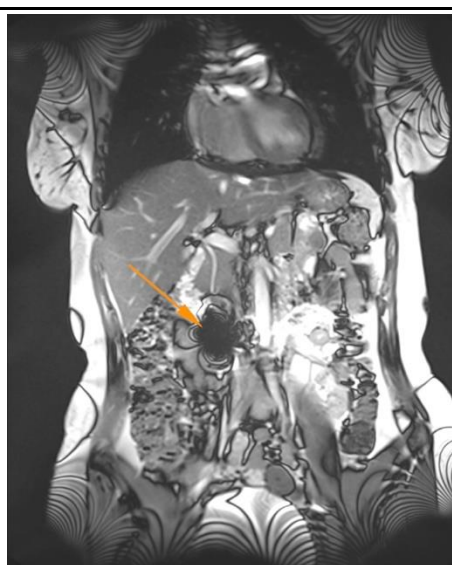

165 min

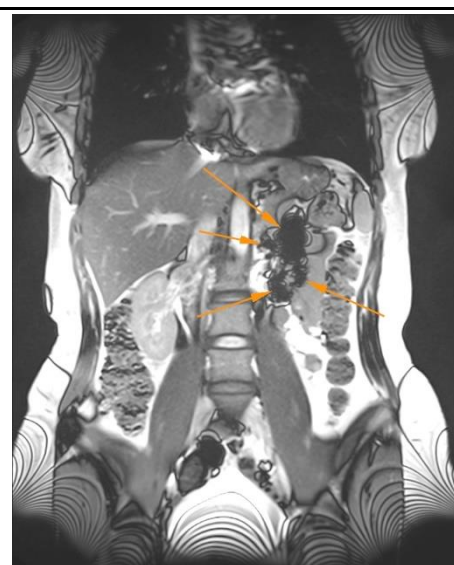

180 min

Supplement: Supplementary file 1 [file pharmaceutics-15-02576-s001.zip › pharmaceutics-2602663 - supplementary file12.pdf]

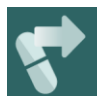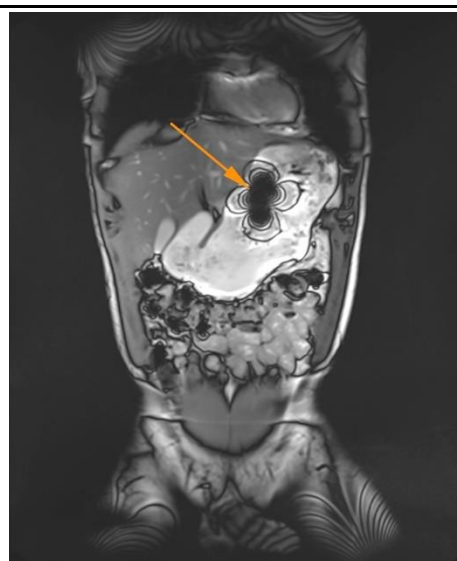

15 min

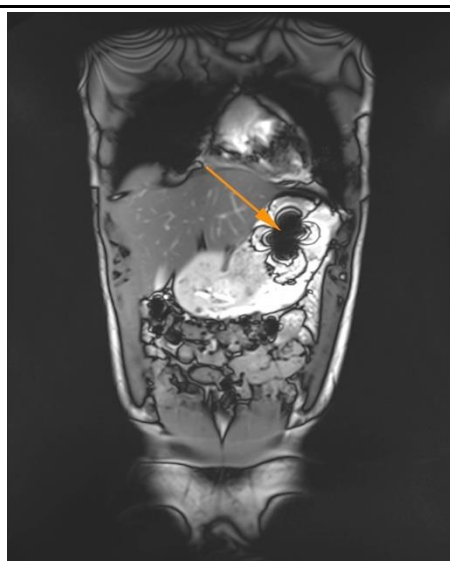

30 min

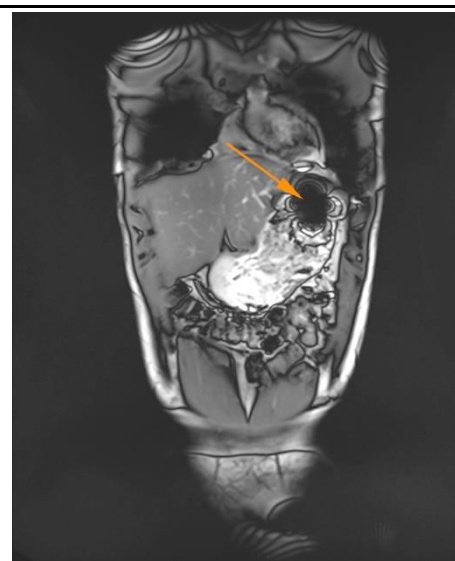

45 min

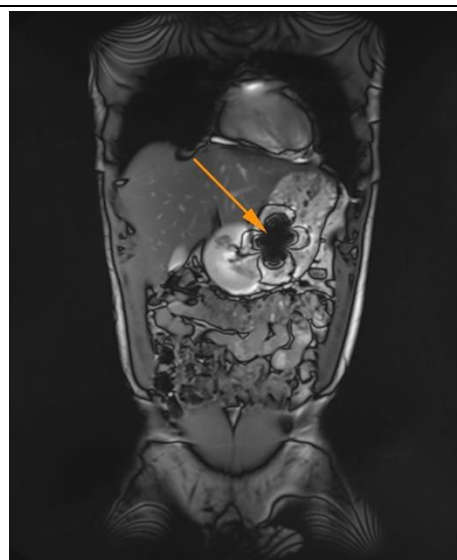

60 min

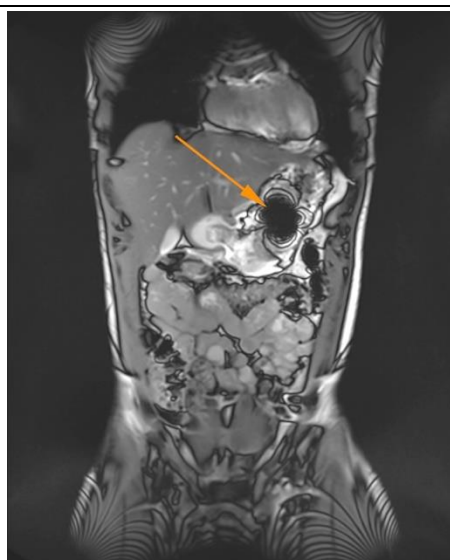

75 min

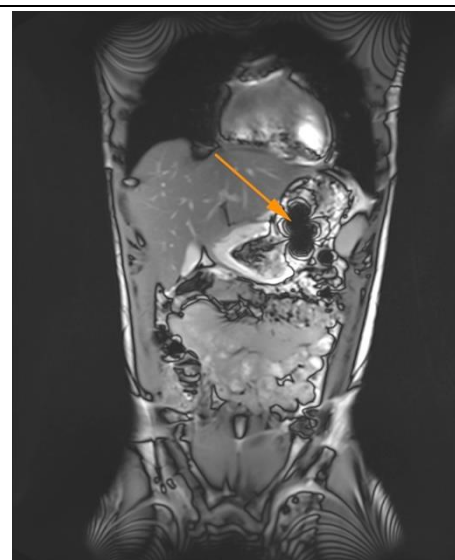

90 min

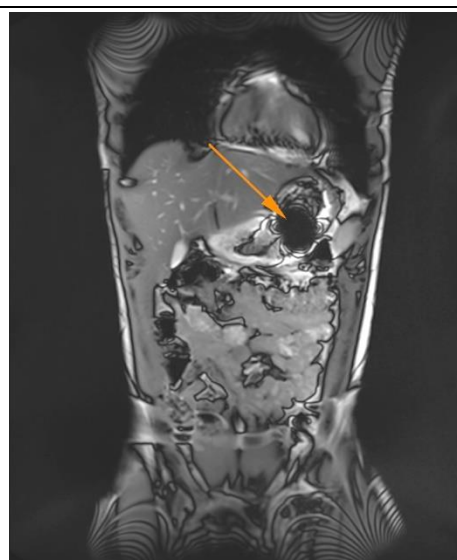

105 min

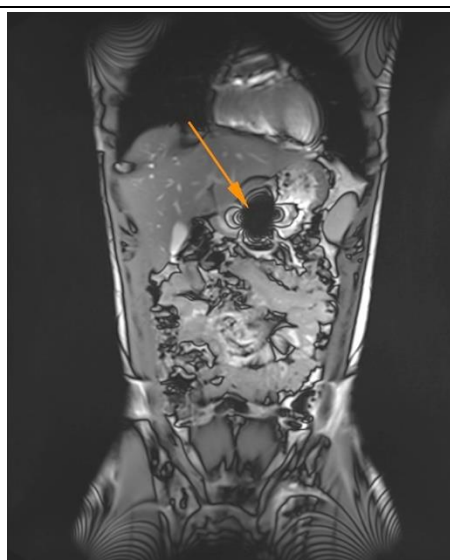

120 min

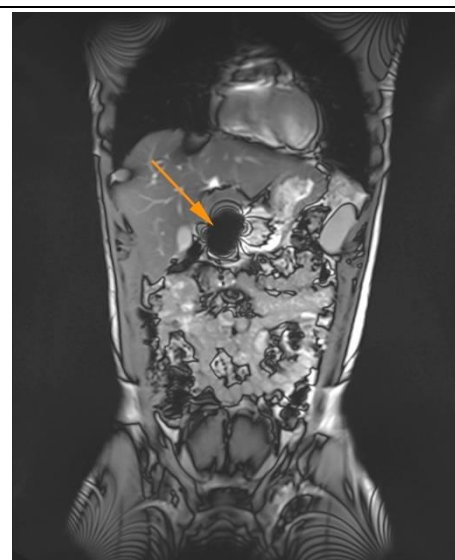

135 min

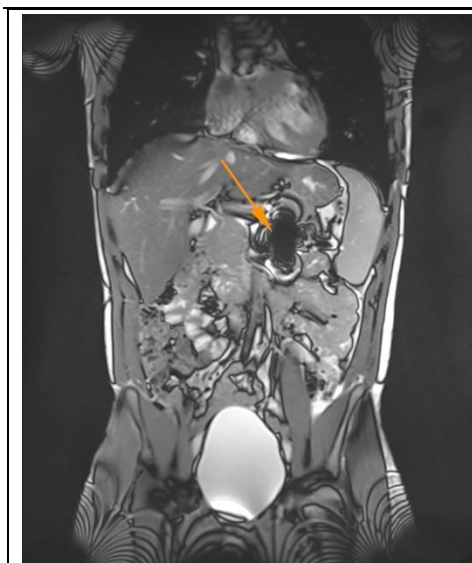

150 min

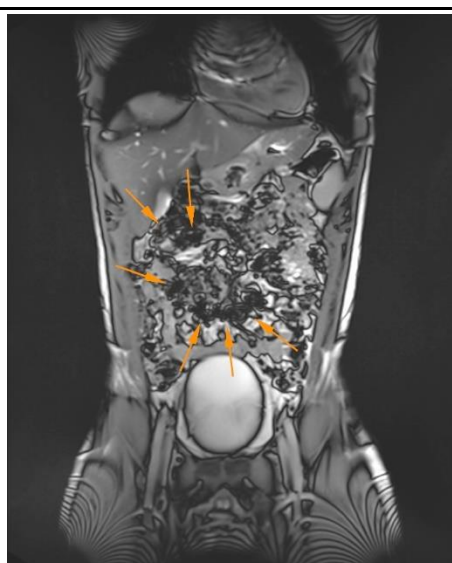

165 min

Supplement: Supplementary file 1 [file pharmaceutics-15-02576-s001.zip › pharmaceutics-2602663 - supplementary file13.pdf]

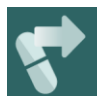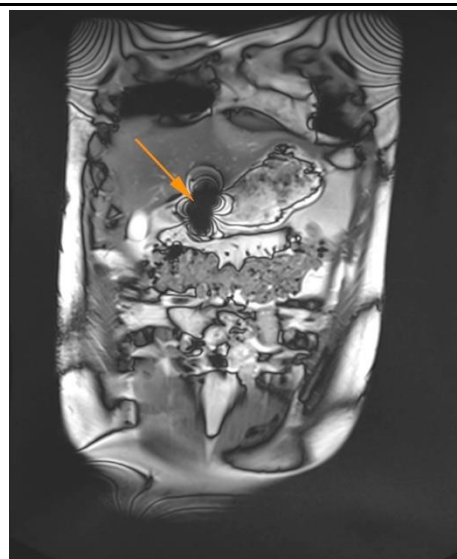

15 min

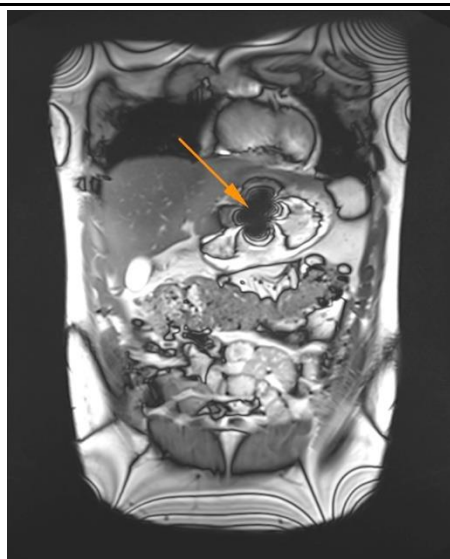

30 min

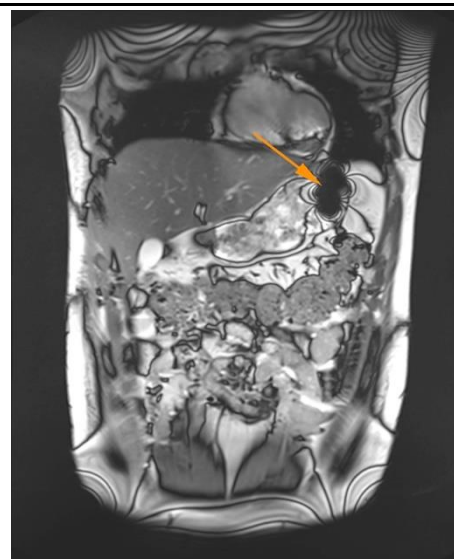

45 min

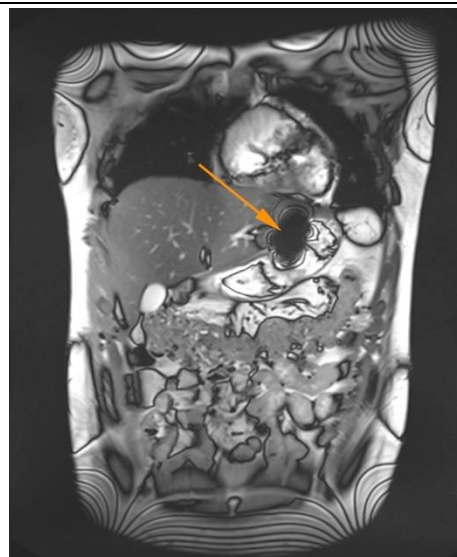

60 min

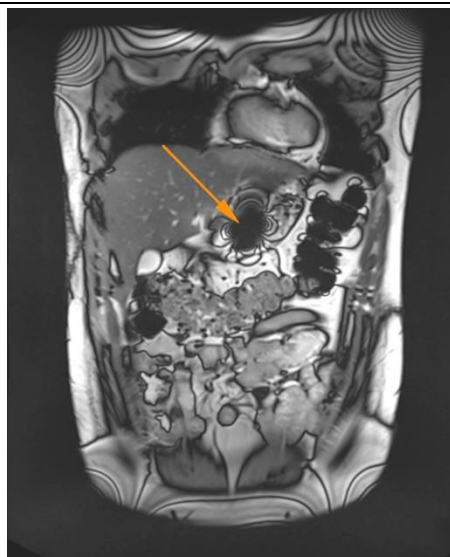

75 min

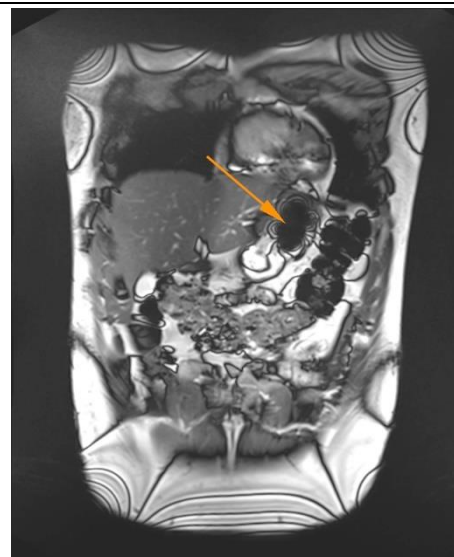

90 min

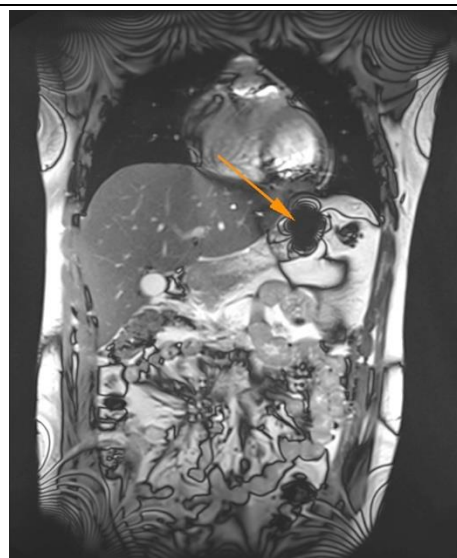

105 min

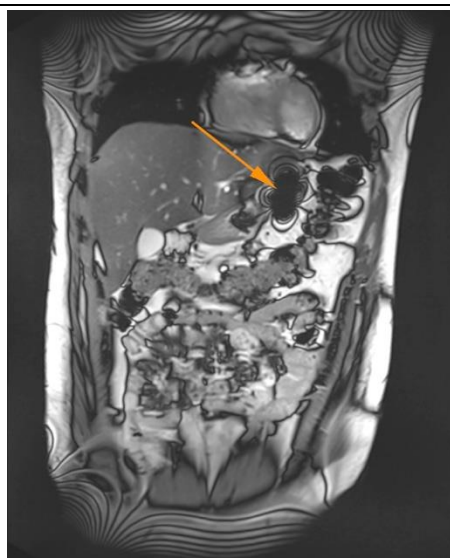

120 min

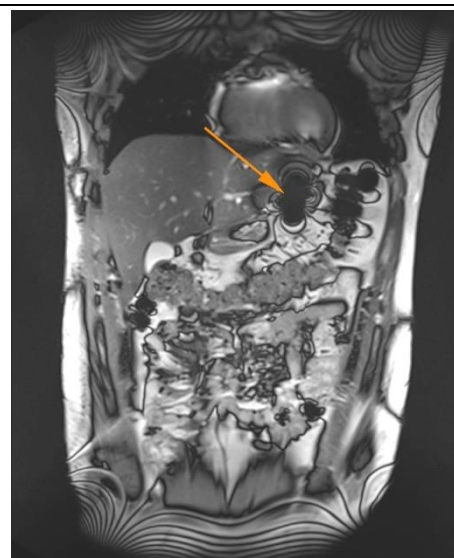

135 min

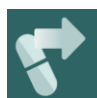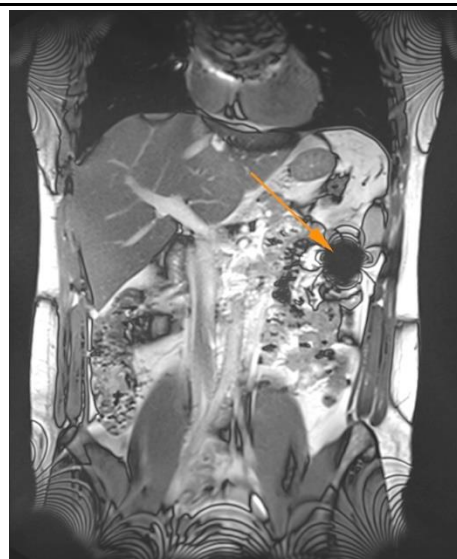

150 min

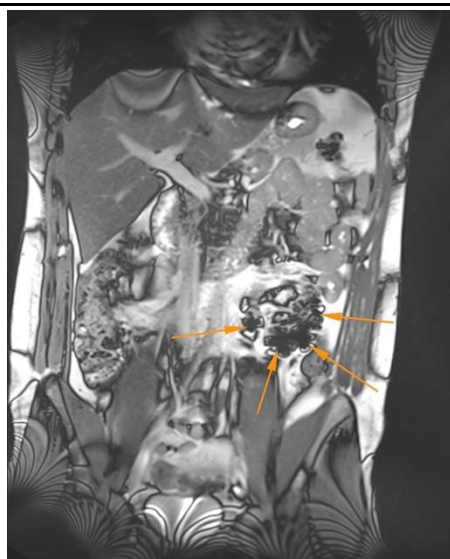

165 min

Supplement: Supplementary file 1 [file pharmaceutics-15-02576-s001.zip › pharmaceutics-2602663 - supplementary file14.pdf]

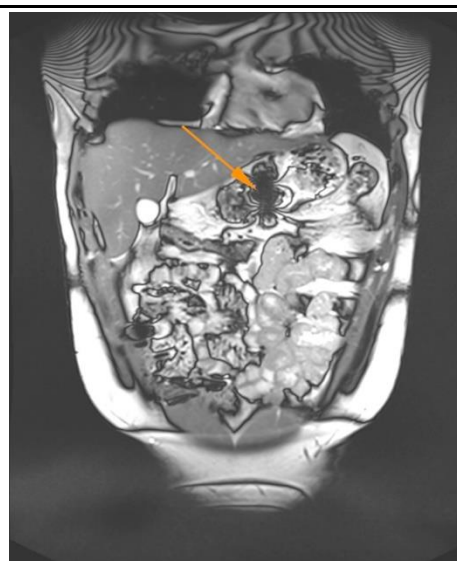

15 min

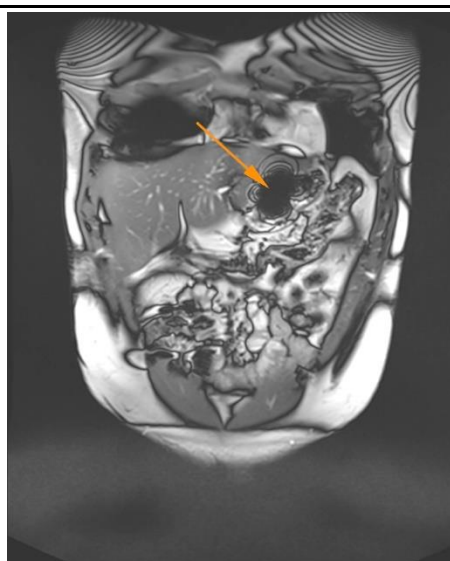

30 min

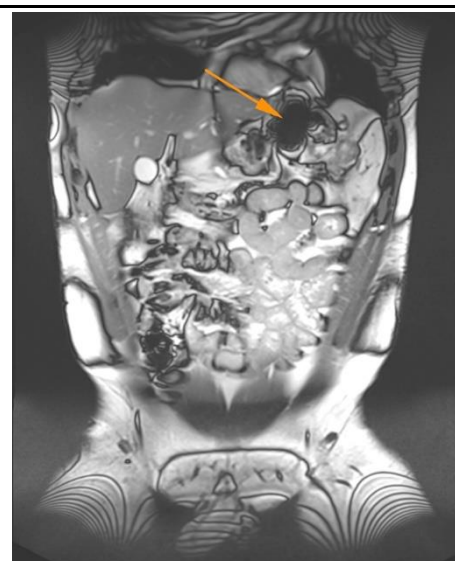

45 min

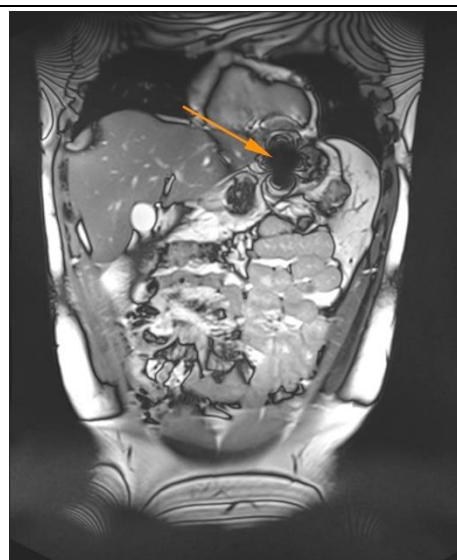

60 min

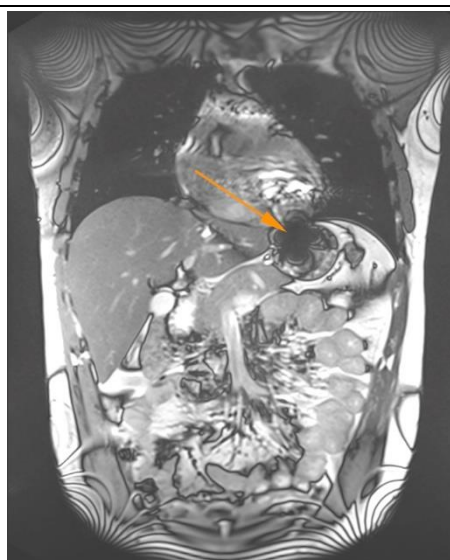

75 min

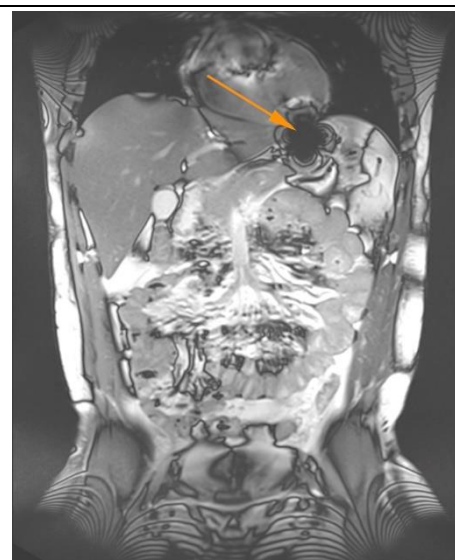

90 min

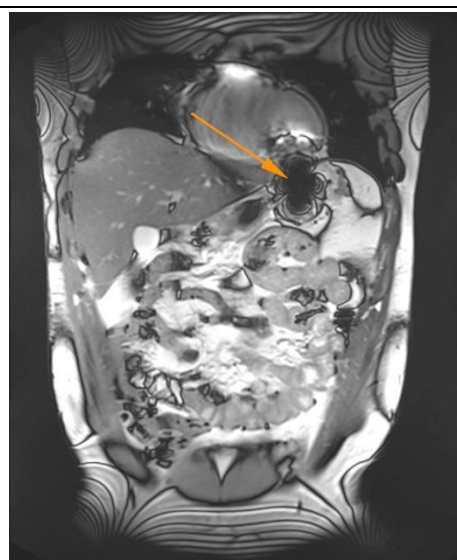

105 min

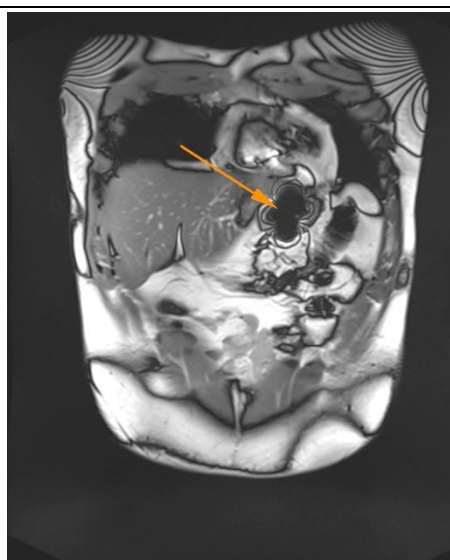

120 min

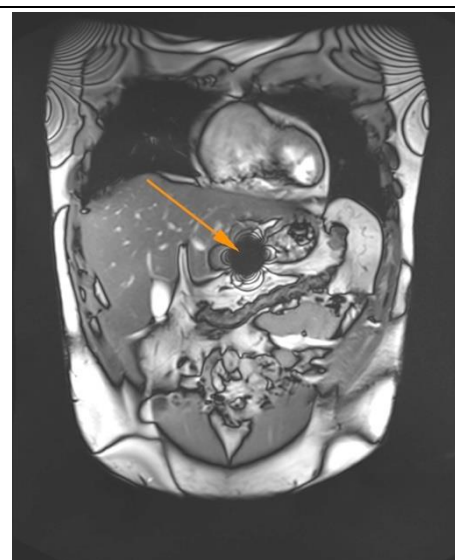

135 min

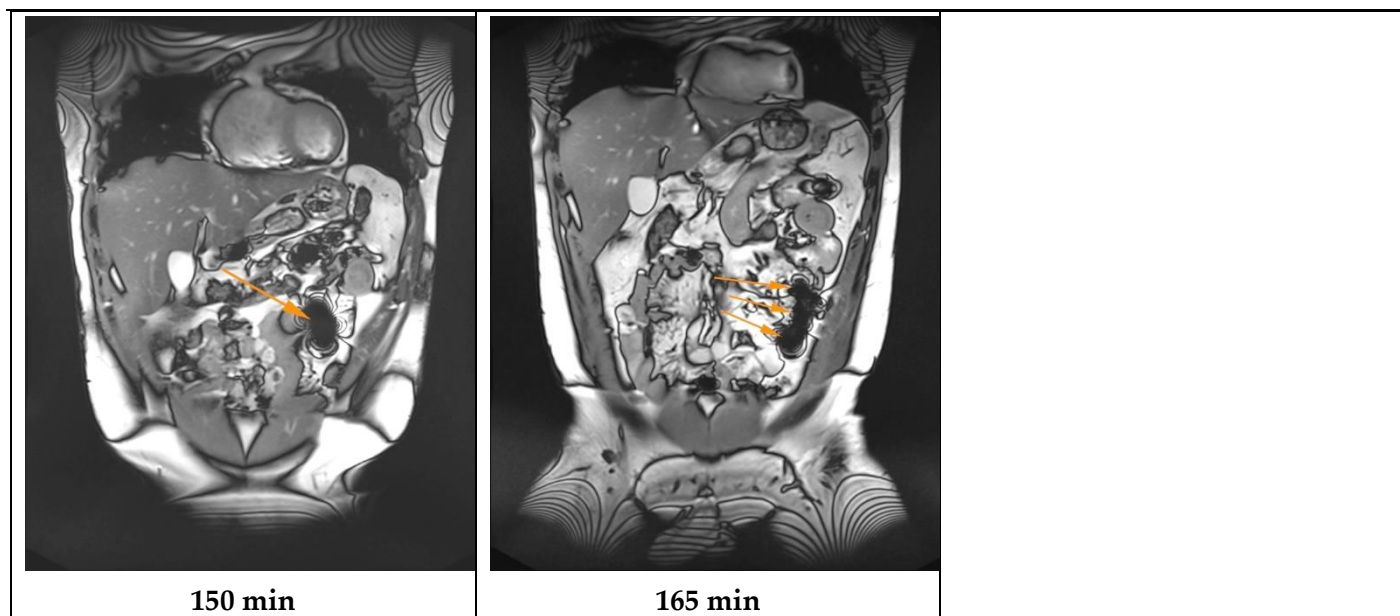

Supplement: Supplementary file 1 [file pharmaceutics-15-02576-s001.zip › pharmaceutics-2602663 - supplementary file15.pdf]

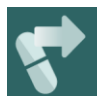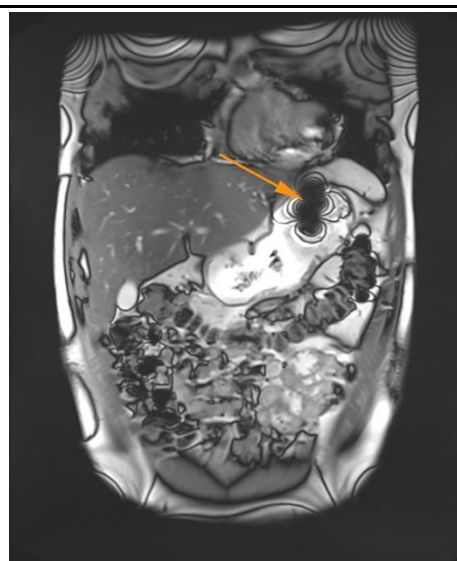

15 min

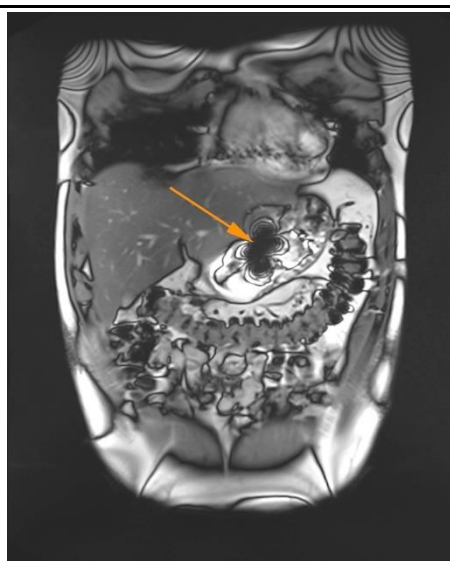

30 min

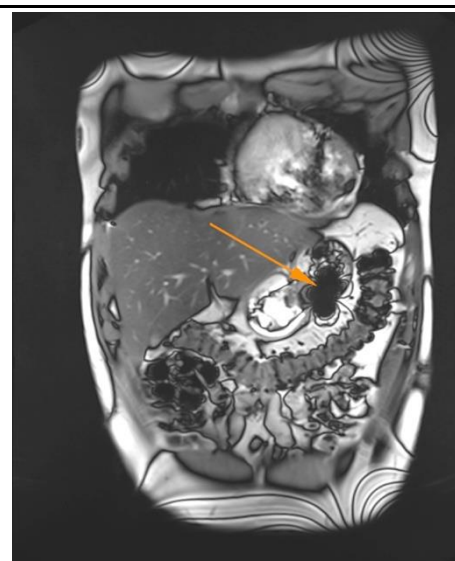

45 min

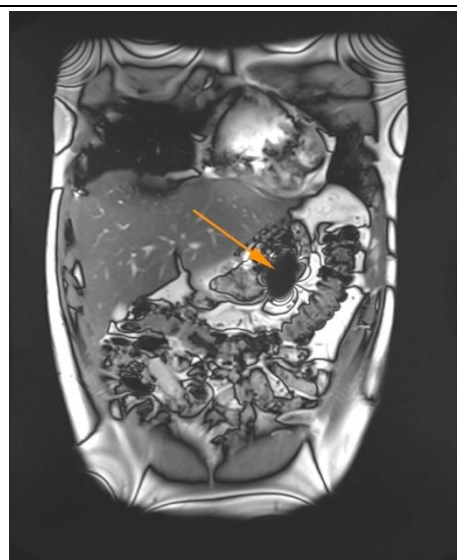

60 min

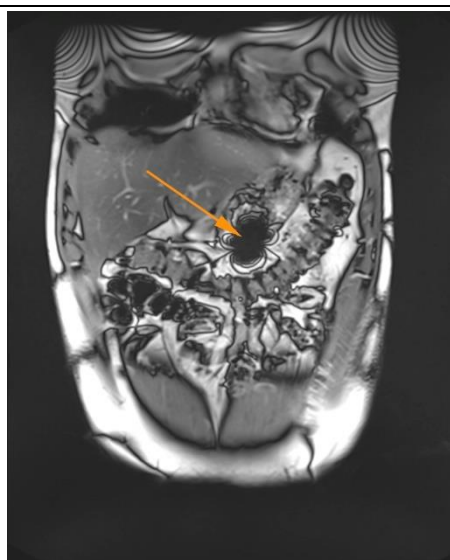

75 min

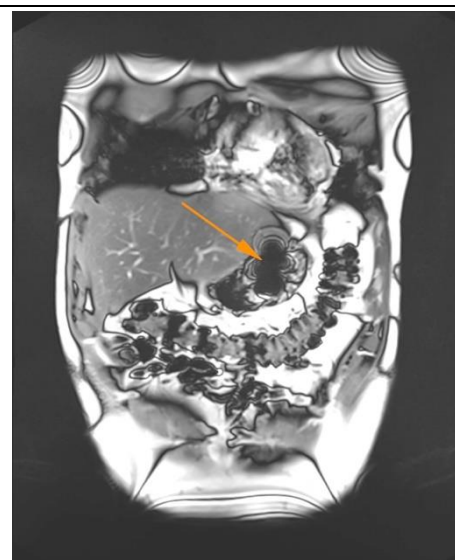

90 min

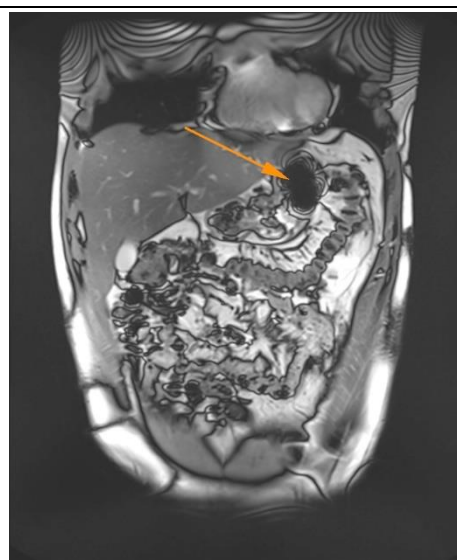

105 min

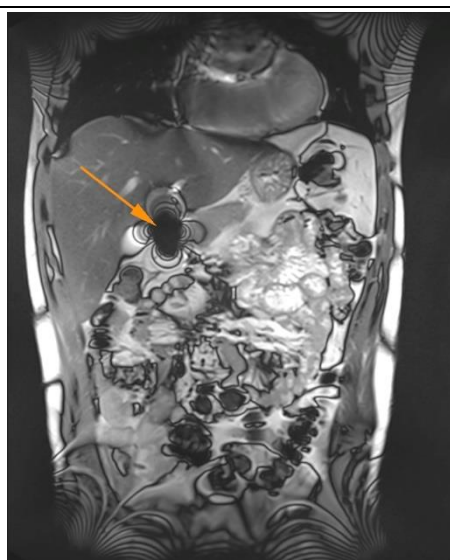

120 min

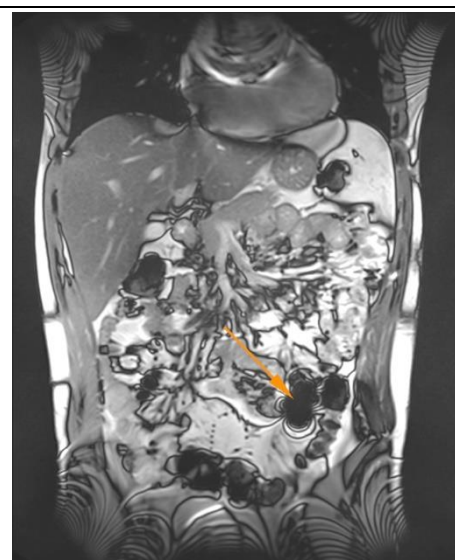

135 min

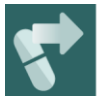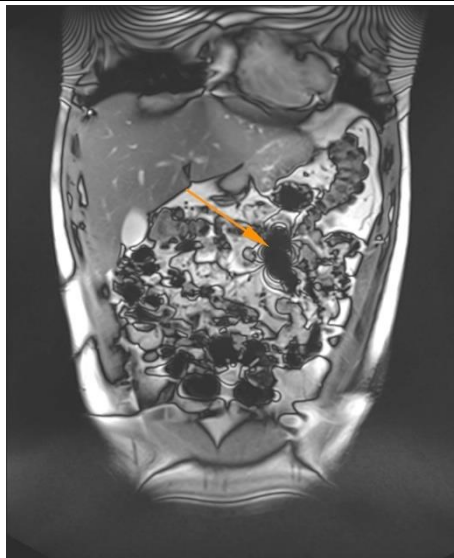

150 min

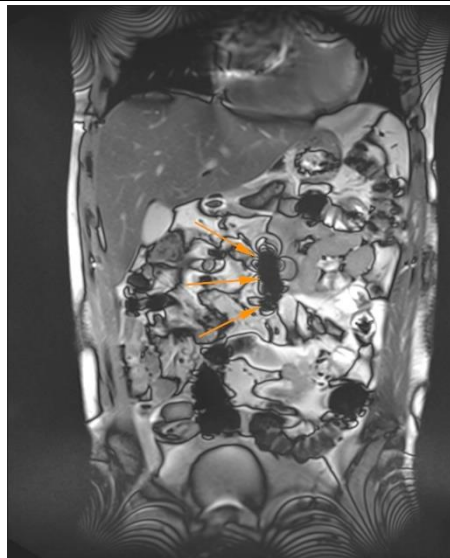

165 min

Supplement: Supplementary file 1 [file pharmaceutics-15-02576-s001.zip › pharmaceutics-2602663 - supplementary file16.pdf]

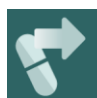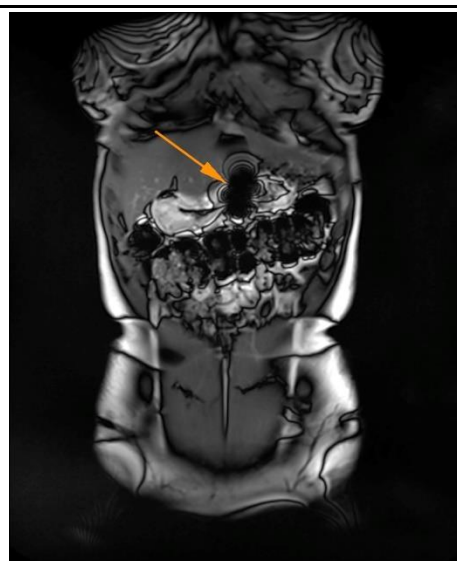

15 min

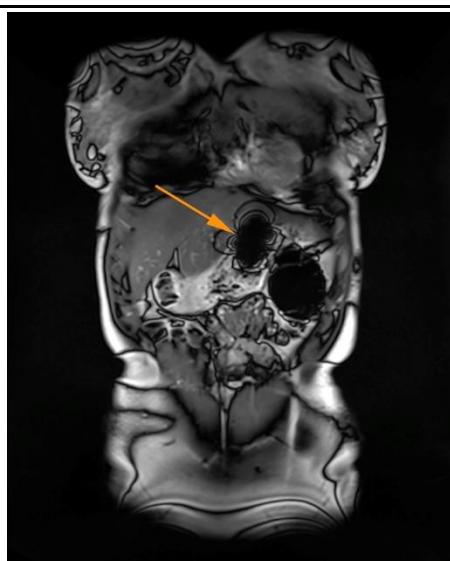

30 min

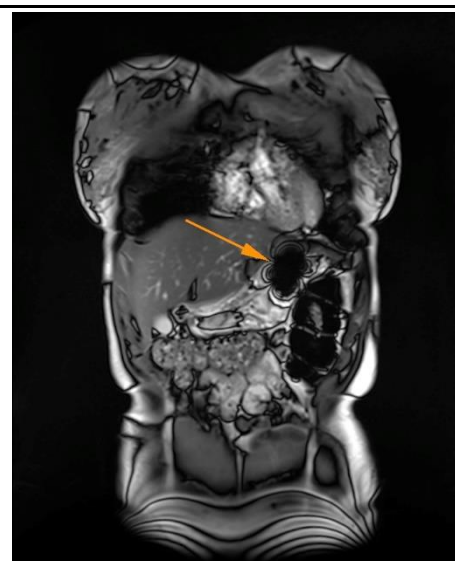

45 min

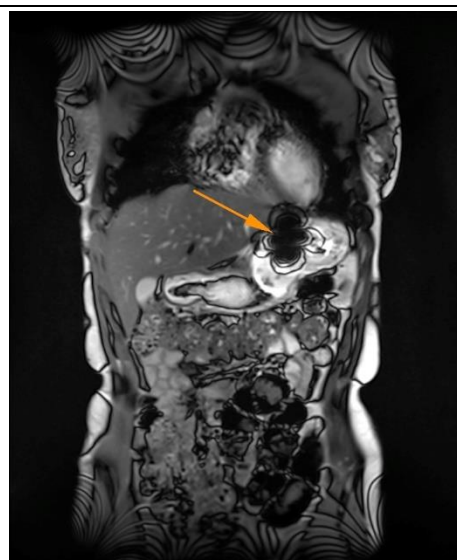

60 min

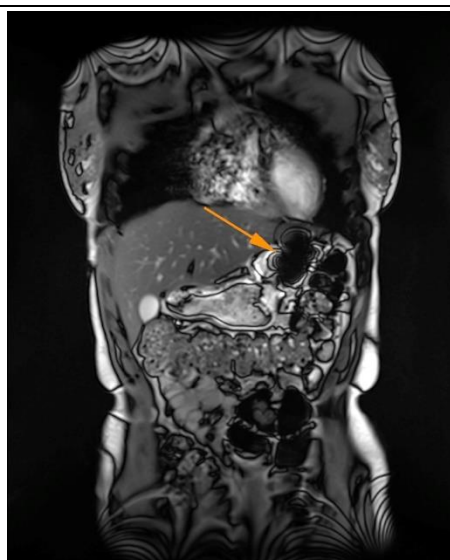

75 min

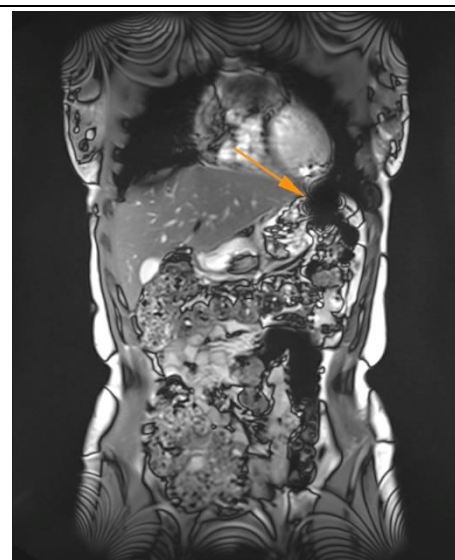

90 min

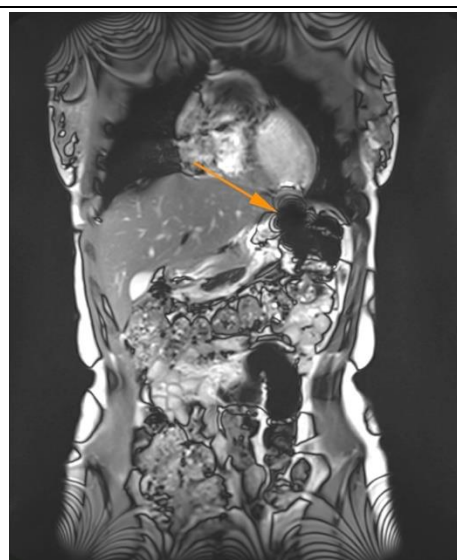

105 min

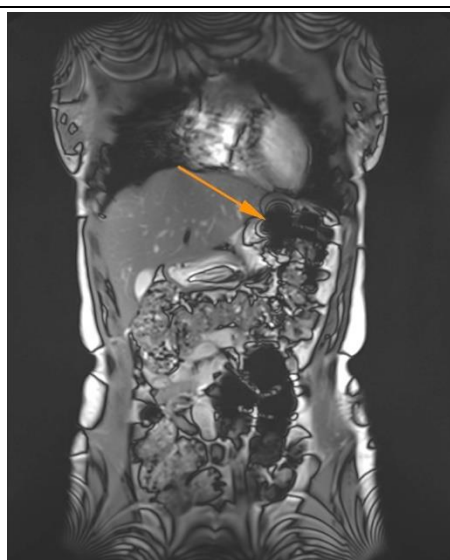

120 min

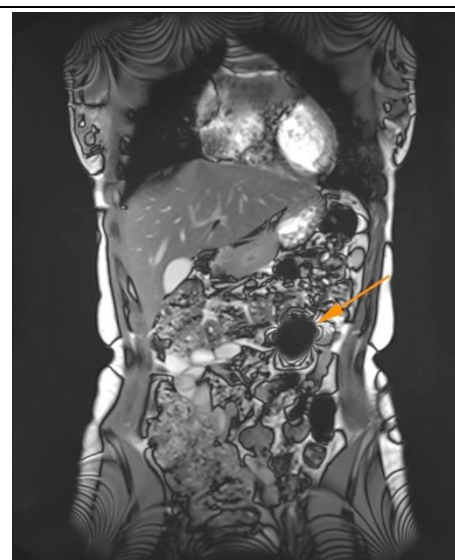

135 min

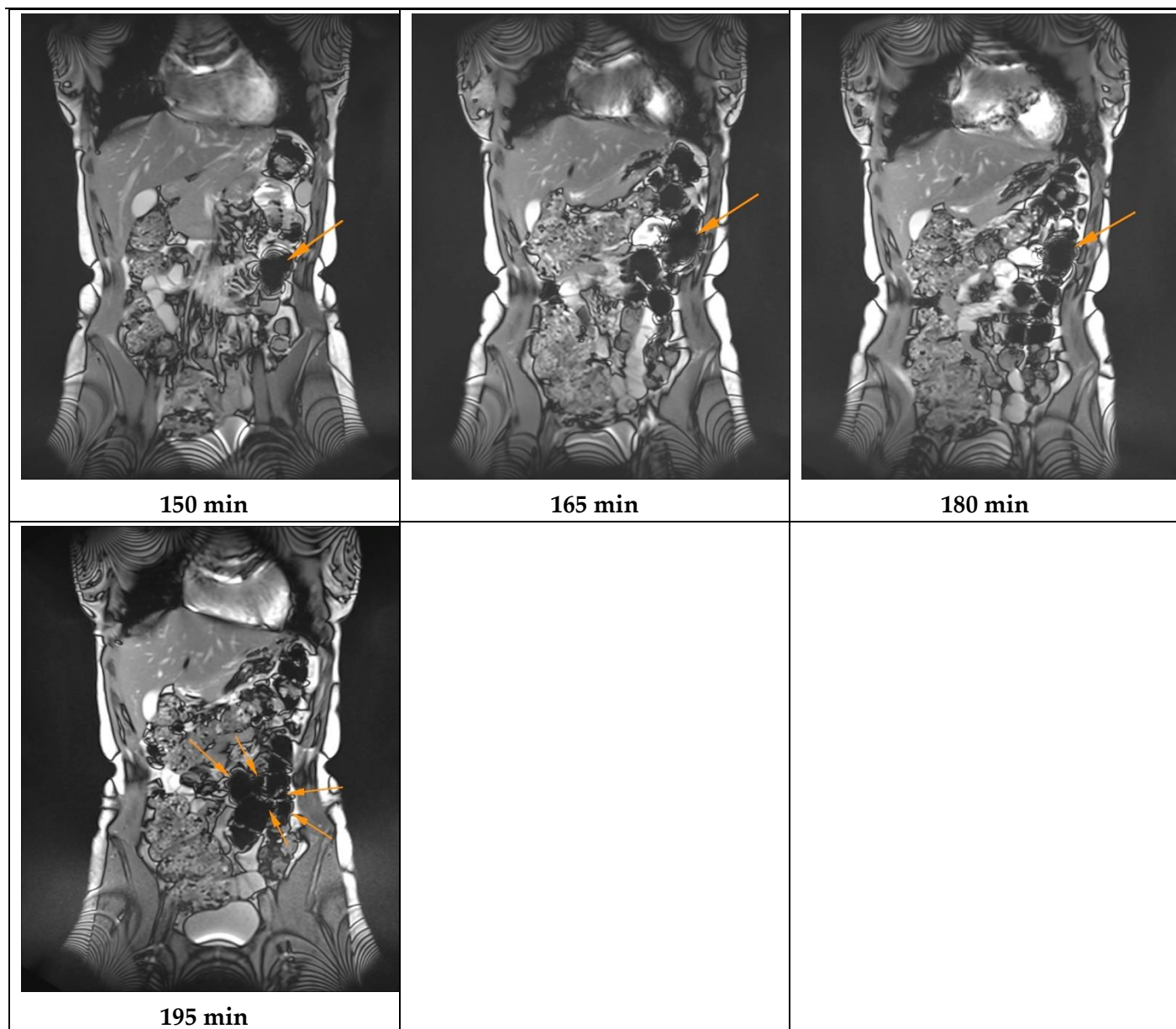

Supplement: Supplementary file 1 [file pharmaceutics-15-02576-s001.zip › pharmaceutics-2602663 - supplementary file2.pdf]

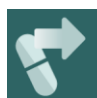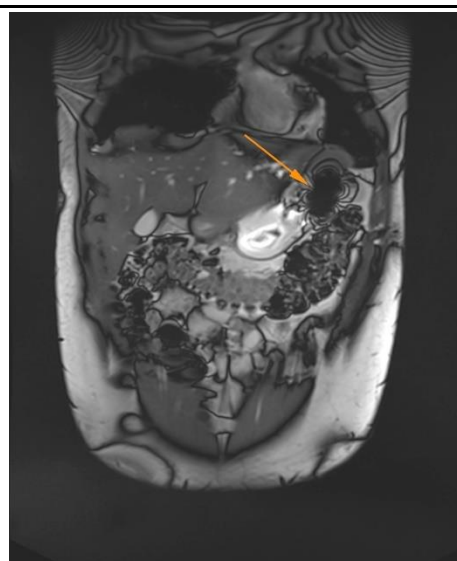

15 min

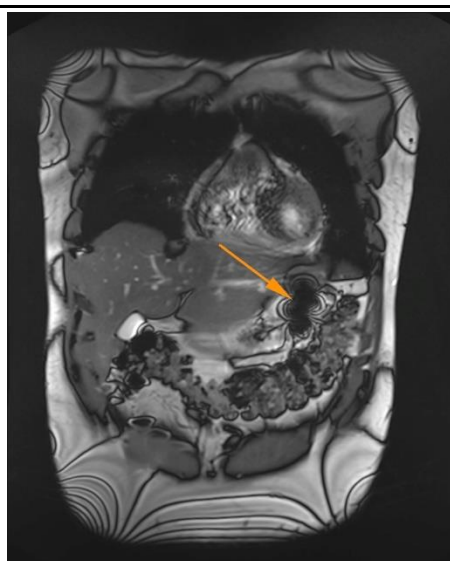

30 min

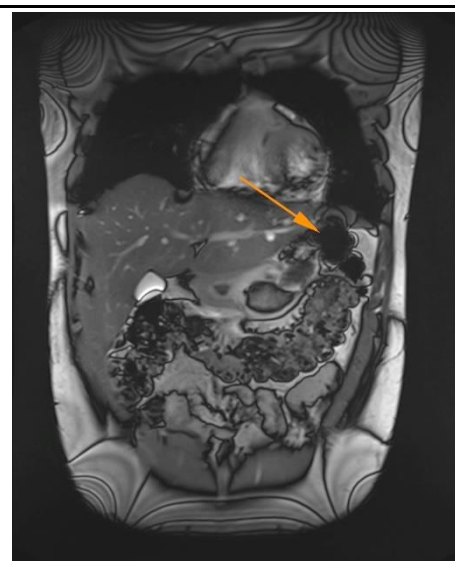

45 min

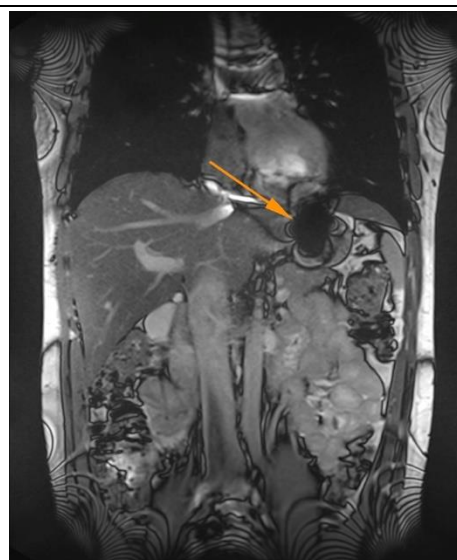

60 min

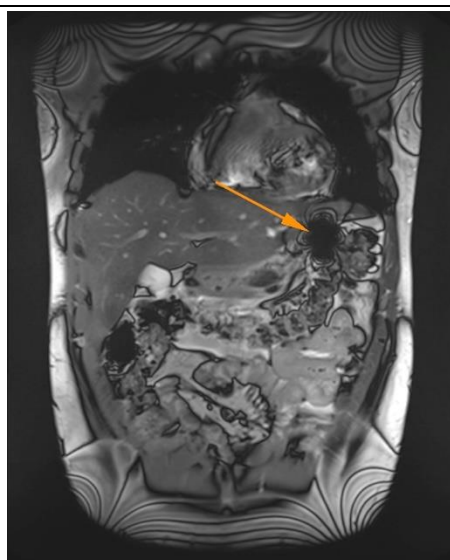

75 min

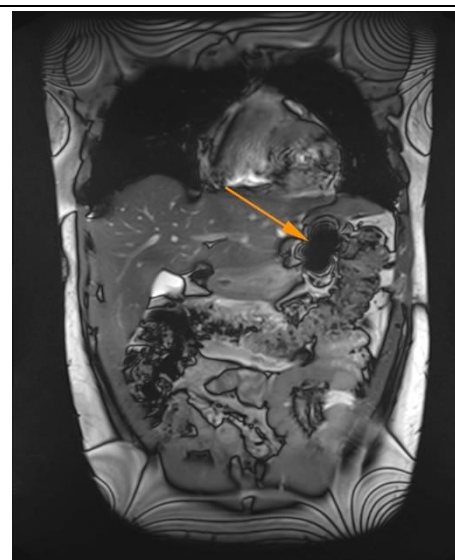

90 min

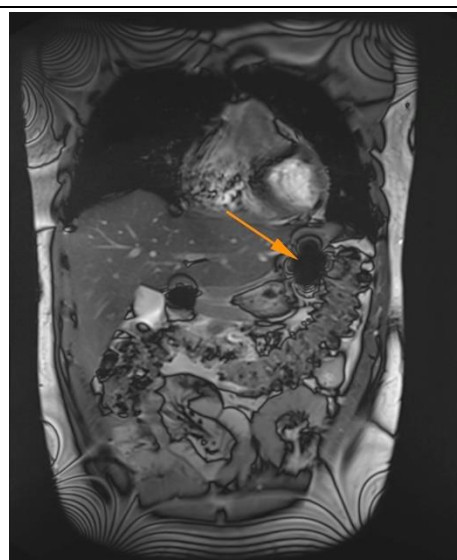

105 min

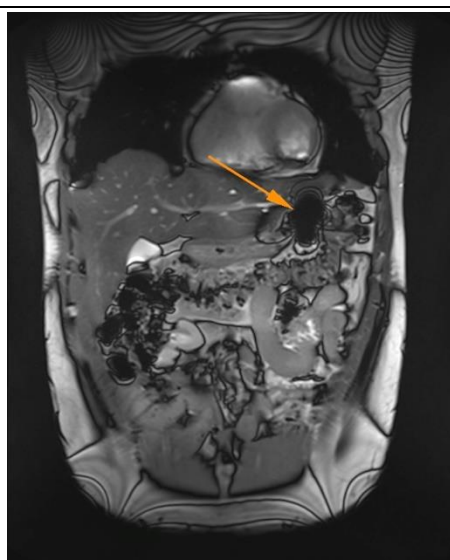

120 min

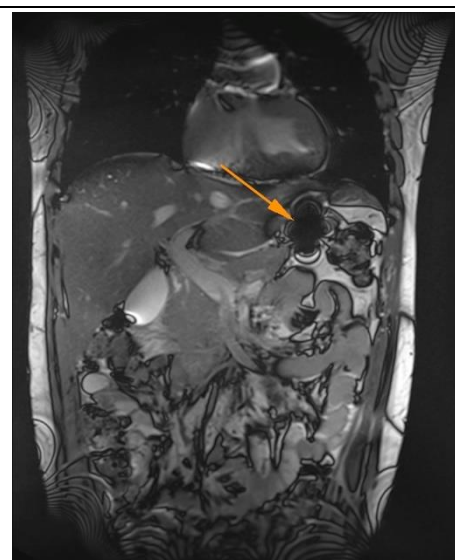

135 min

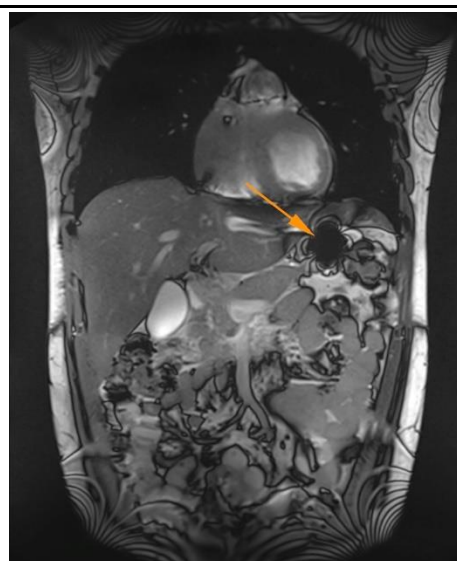

150 min

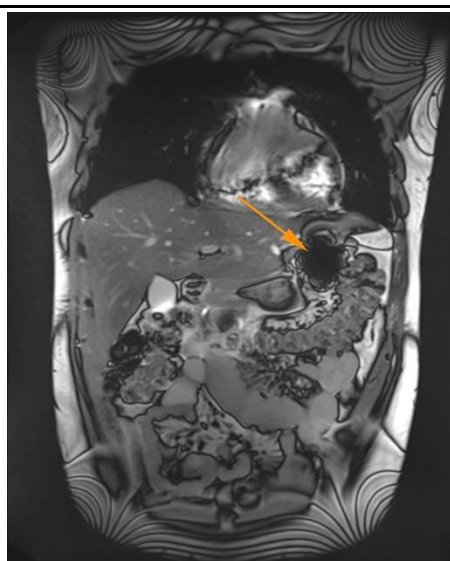

165 min

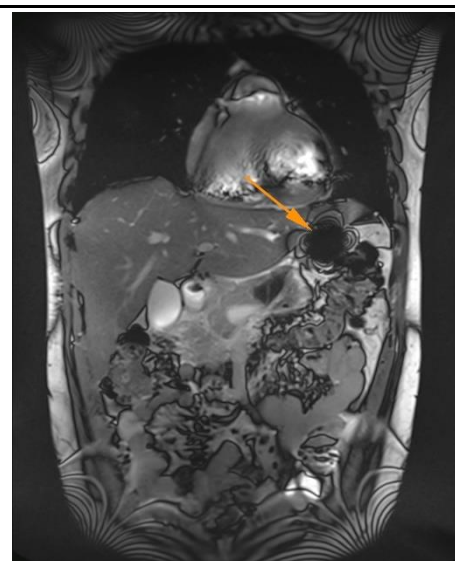

180 min

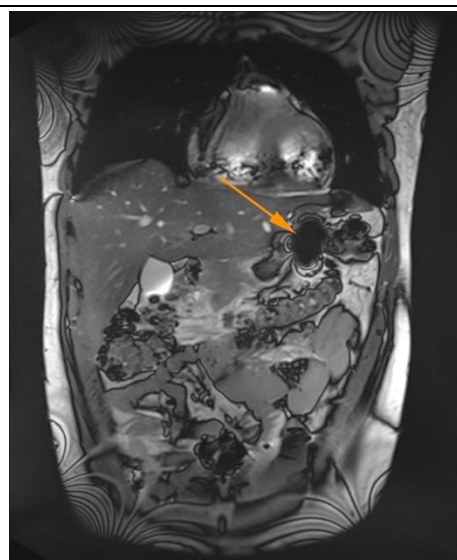

195 min

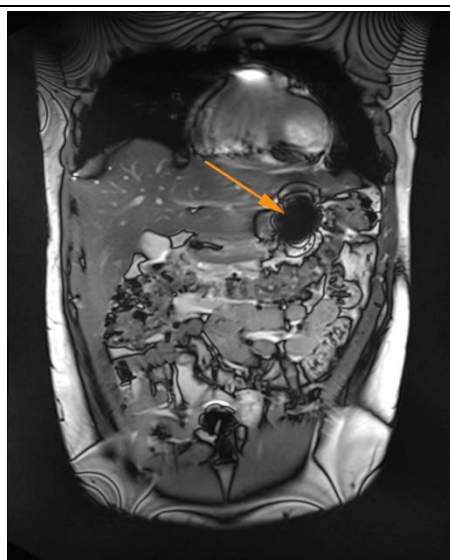

210 min

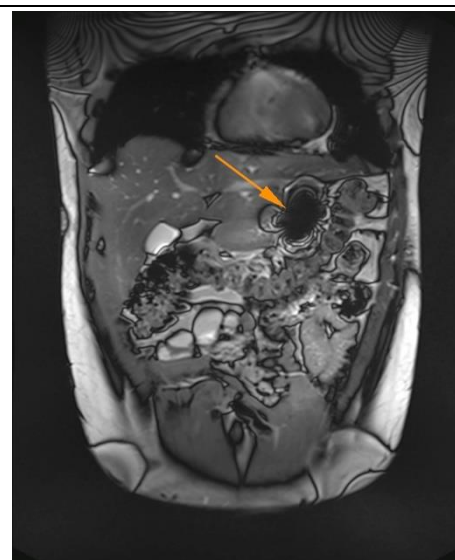

225 min

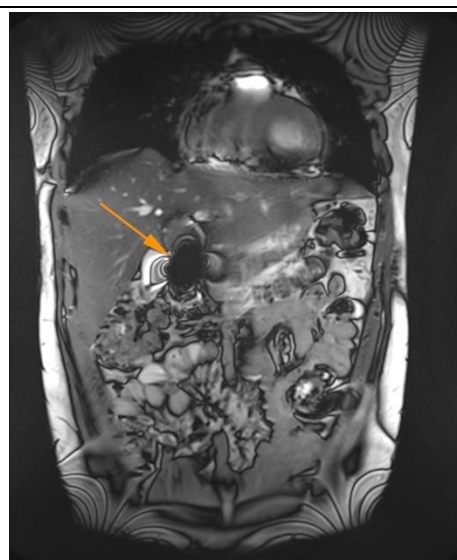

240 min

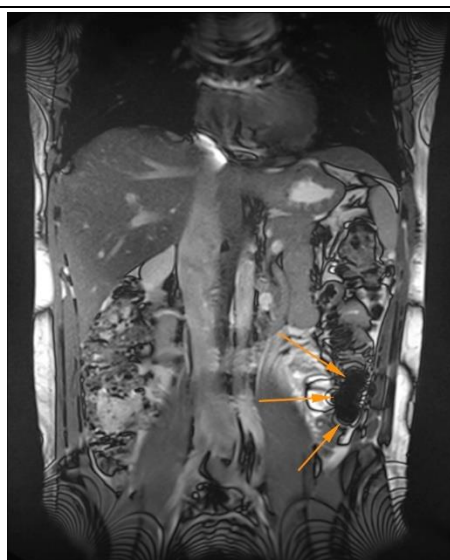

255 min

Supplement: Supplementary file 1 [file pharmaceutics-15-02576-s001.zip › pharmaceutics-2602663 - supplementary file3.pdf]

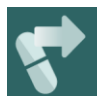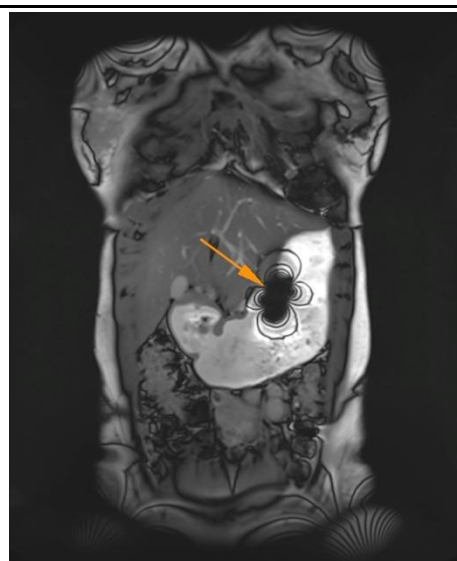

15 min

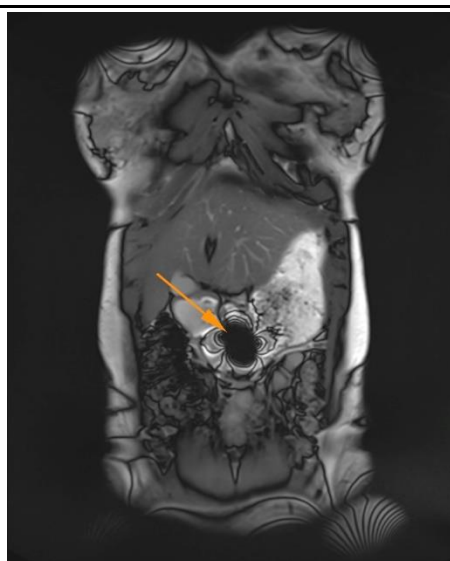

30 min

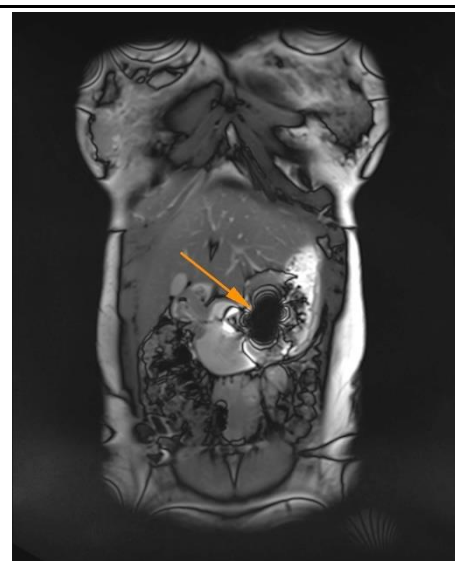

45 min

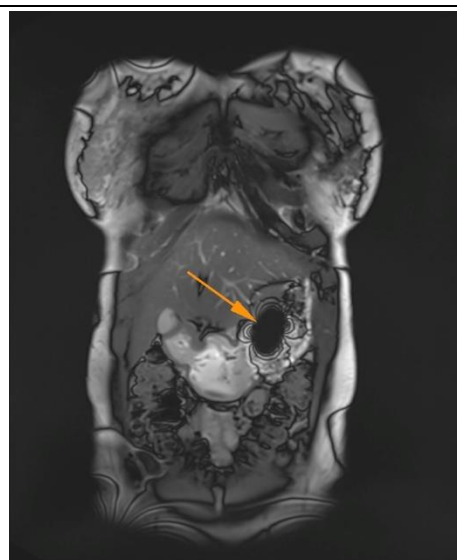

60 min

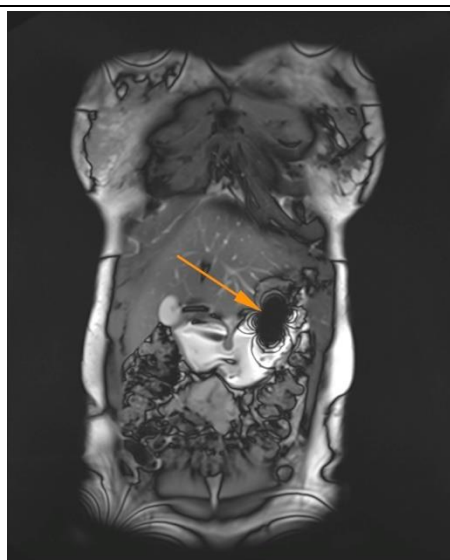

75 min

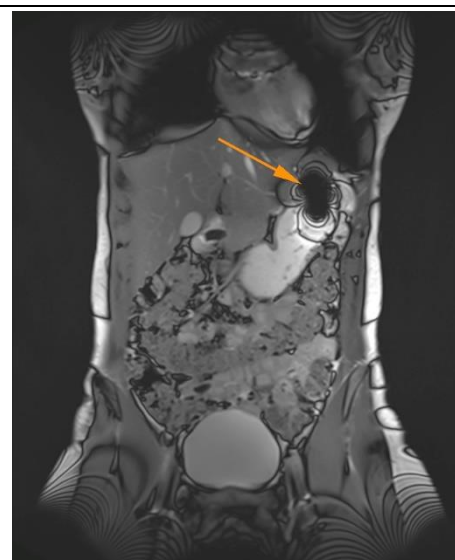

90 min

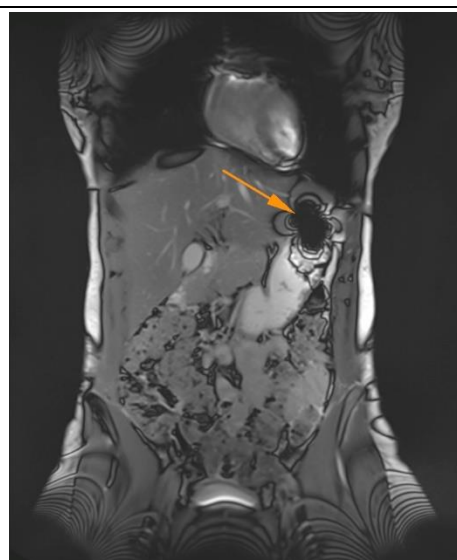

105 min

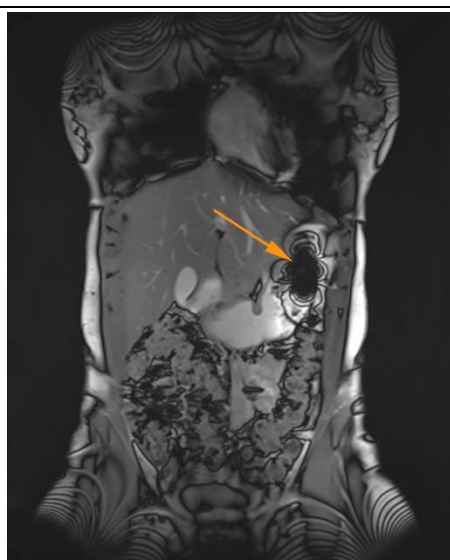

120 min

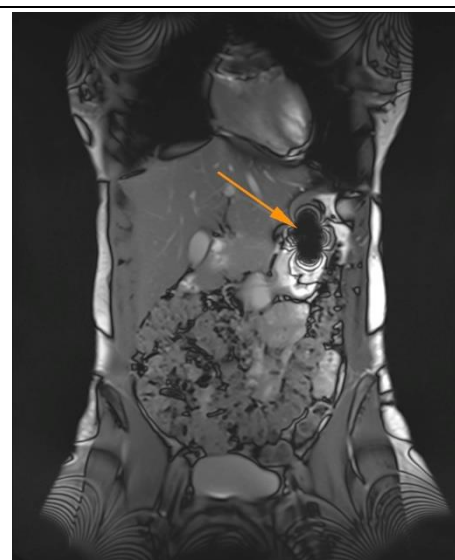

135 min

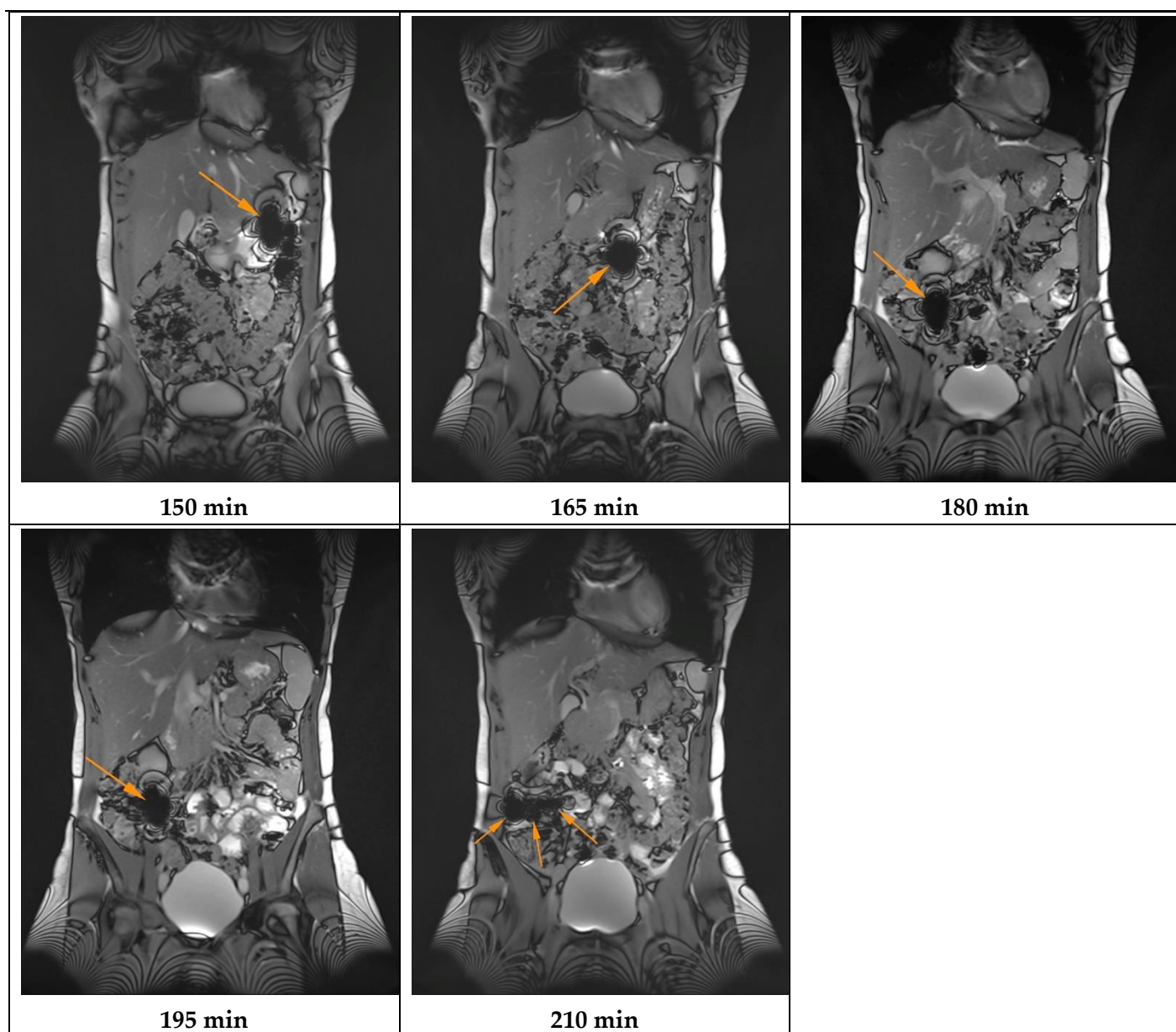

Supplement: Supplementary file 1 [file pharmaceutics-15-02576-s001.zip › pharmaceutics-2602663 - supplementary file4.pdf]

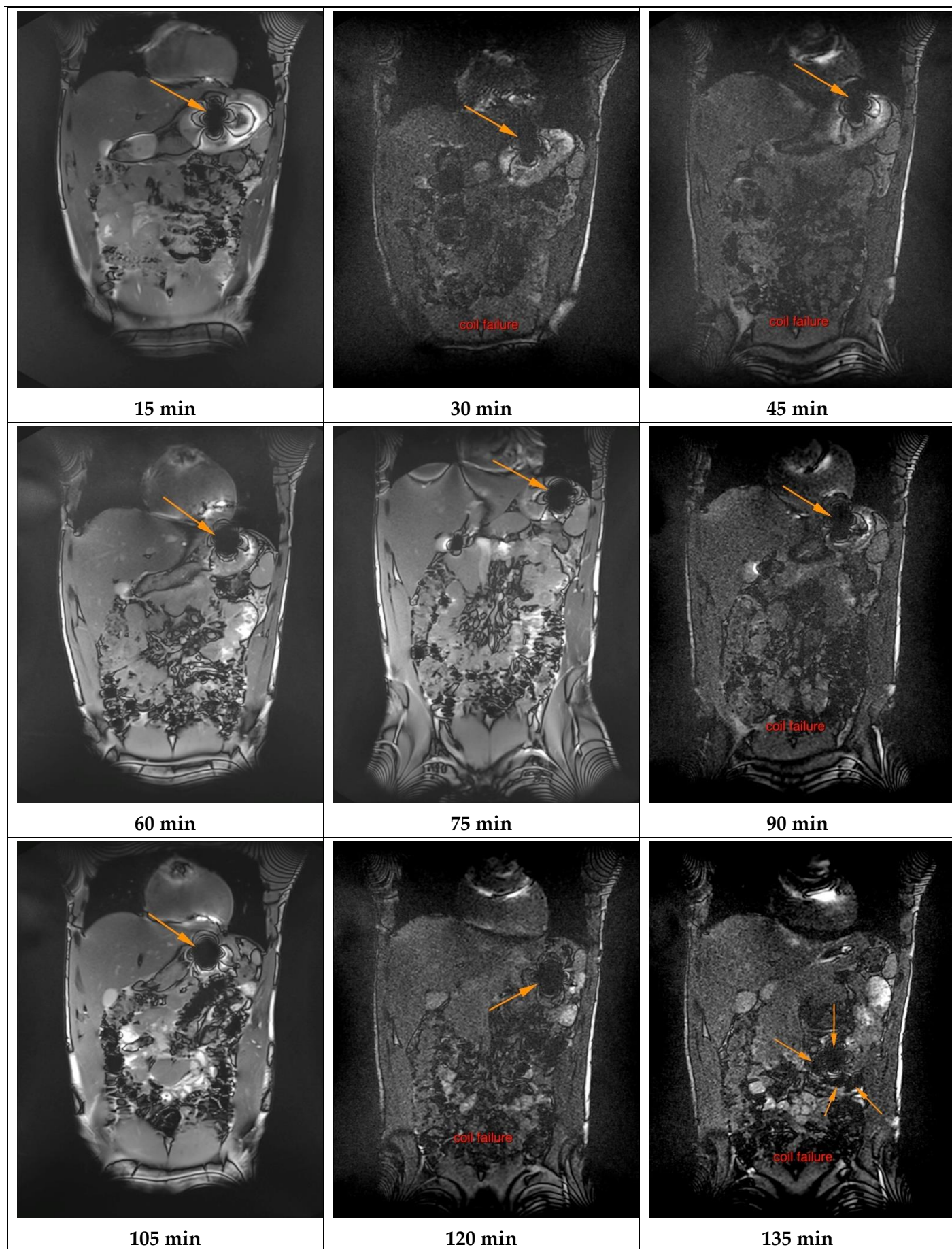

Supplement: Supplementary file 1 [file pharmaceutics-15-02576-s001.zip › pharmaceutics-2602663 - supplementary file5.pdf]

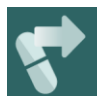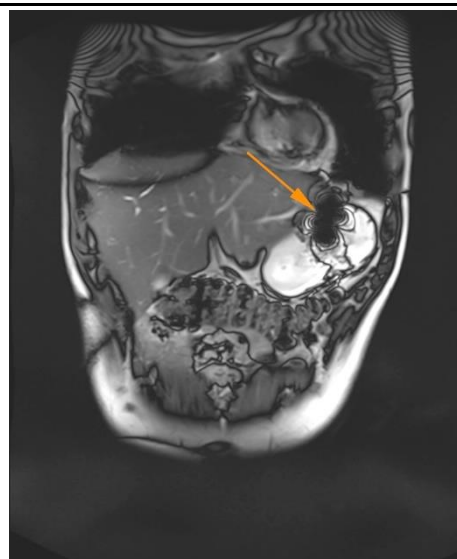

15 min

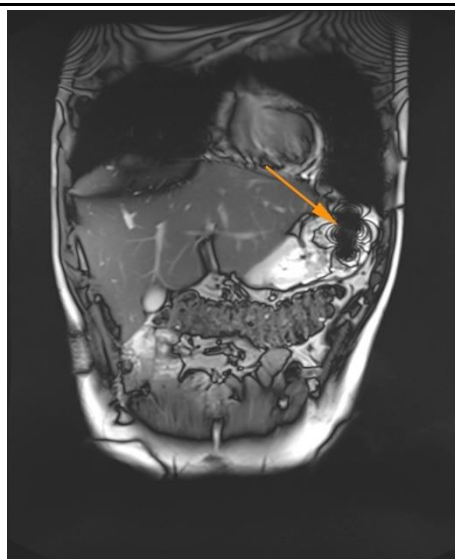

30 min

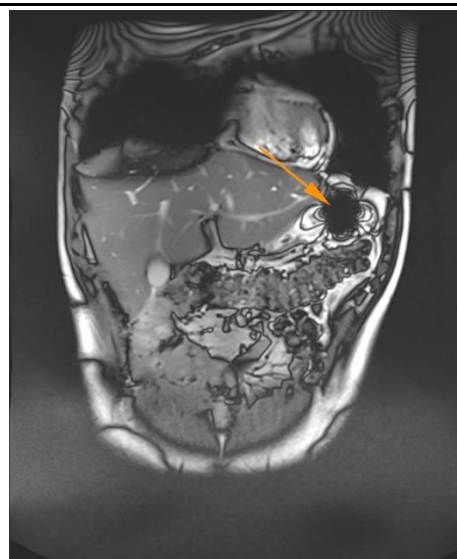

45 min

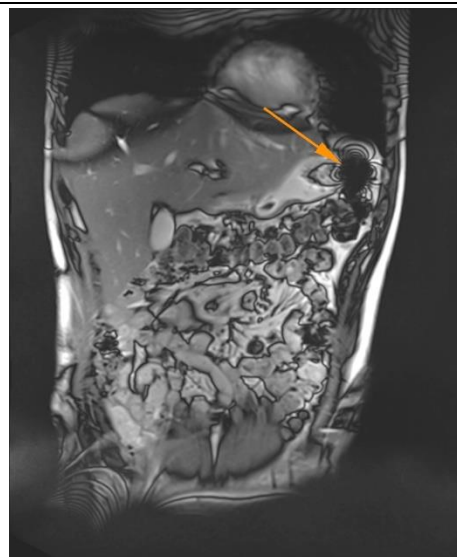

60 min

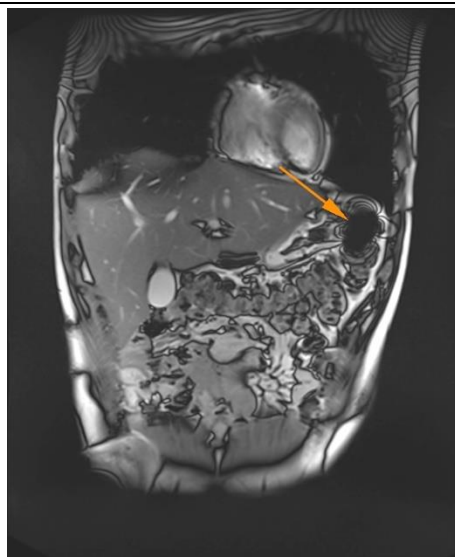

75 min

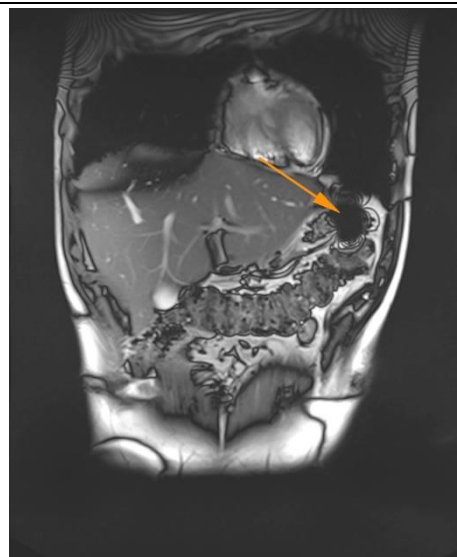

90 min

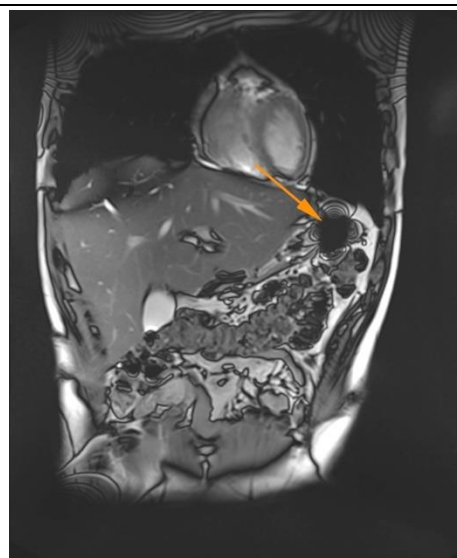

105 min

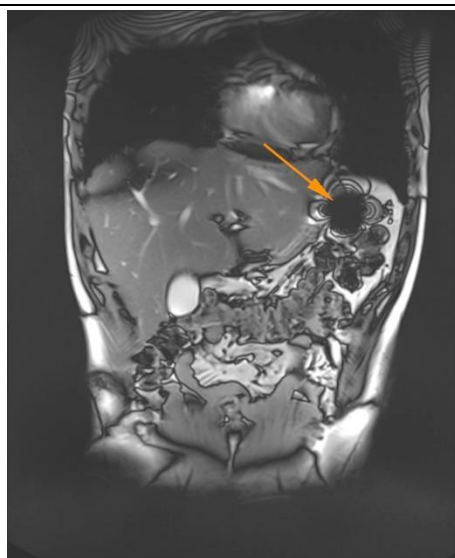

120 min

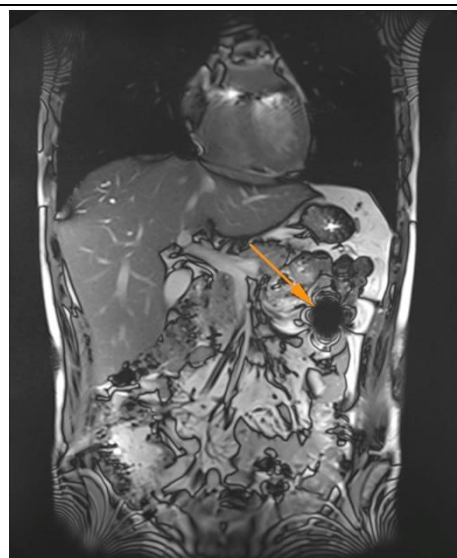

135 min

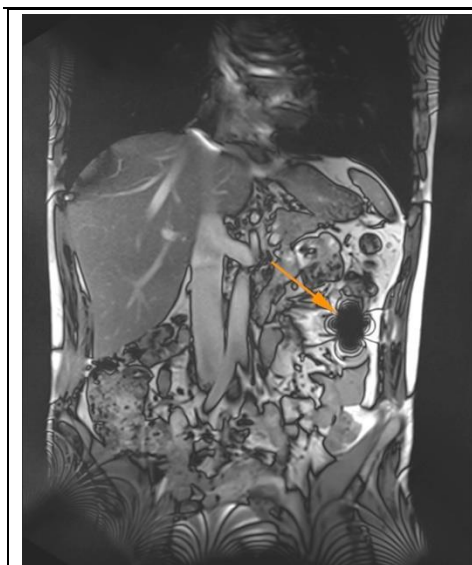

150 min

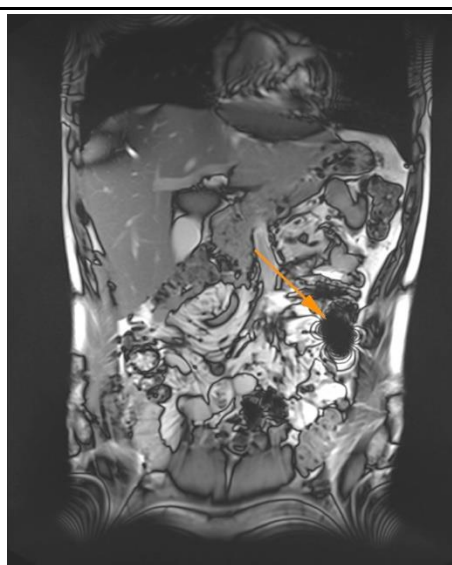

165 min

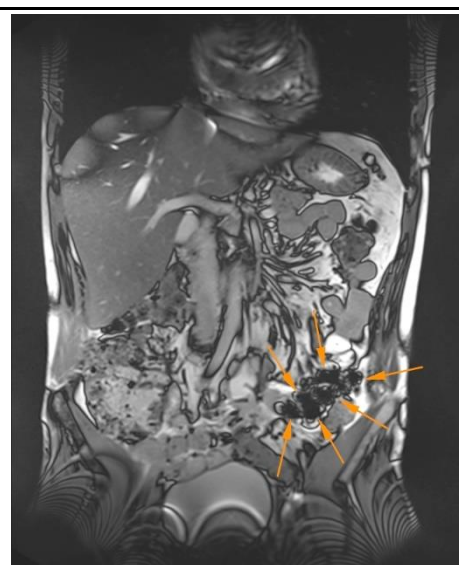

180 min

Supplement: Supplementary file 1 [file pharmaceutics-15-02576-s001.zip › pharmaceutics-2602663 - supplementary file6.pdf]

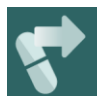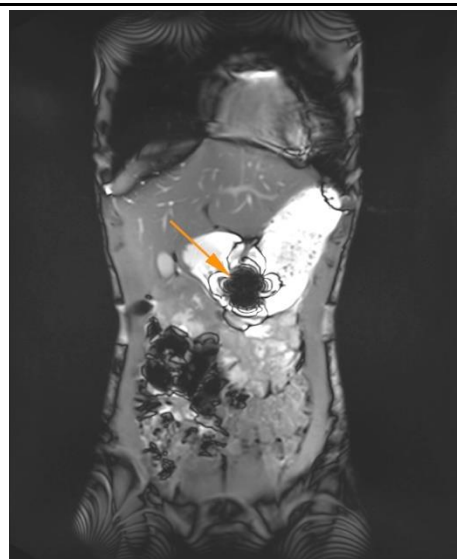

15 min

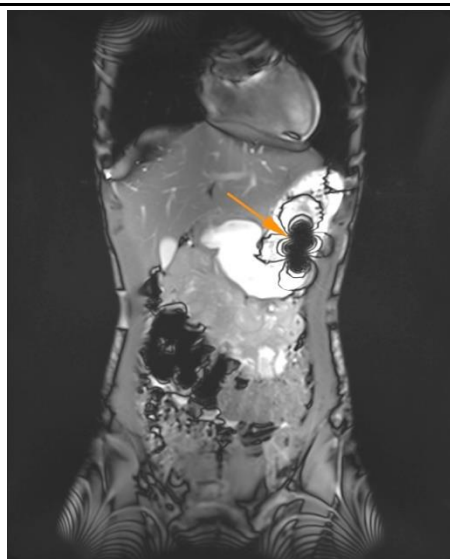

30 min

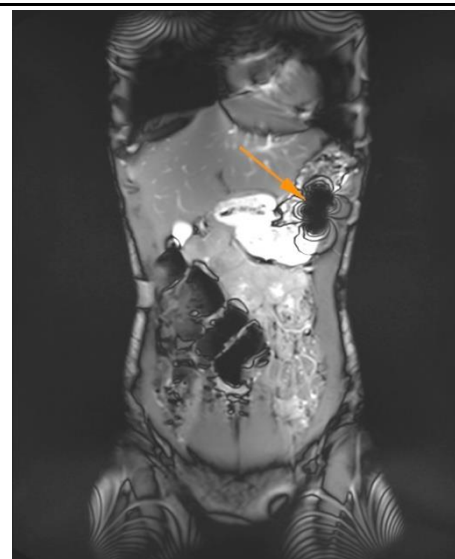

45 min

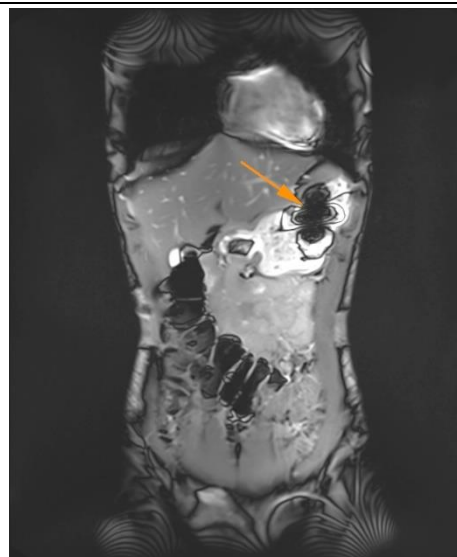

60 min

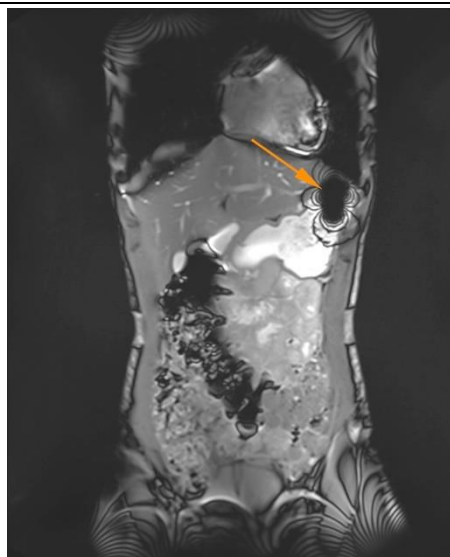

75 min

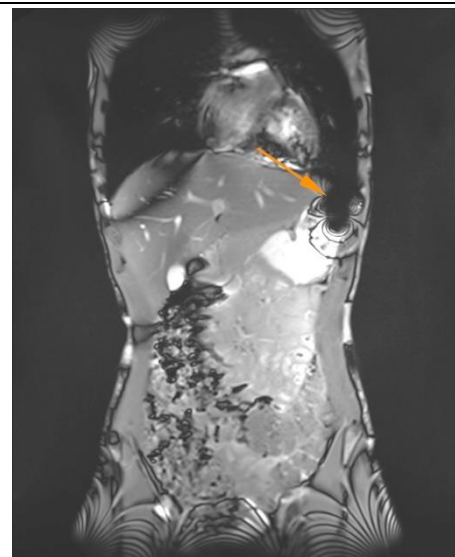

90 min

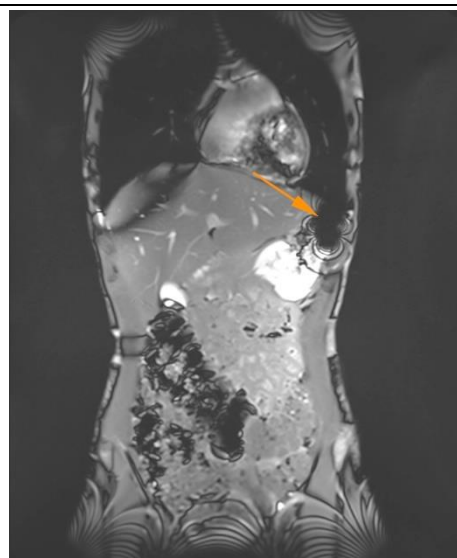

105 min

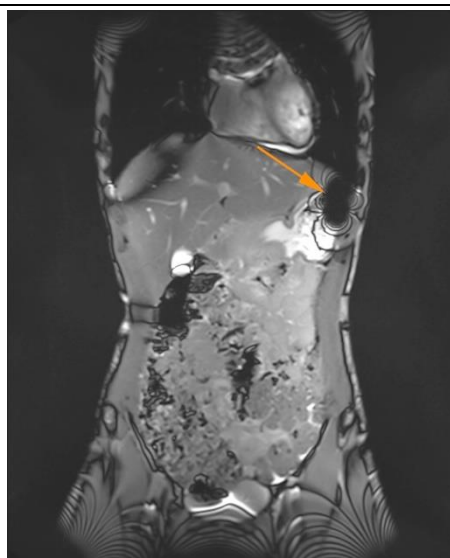

120 min

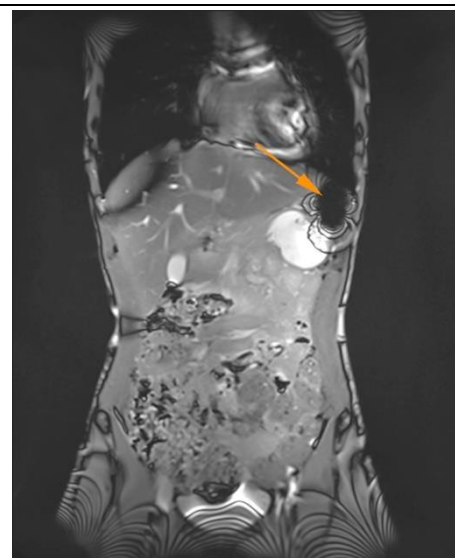

135 min

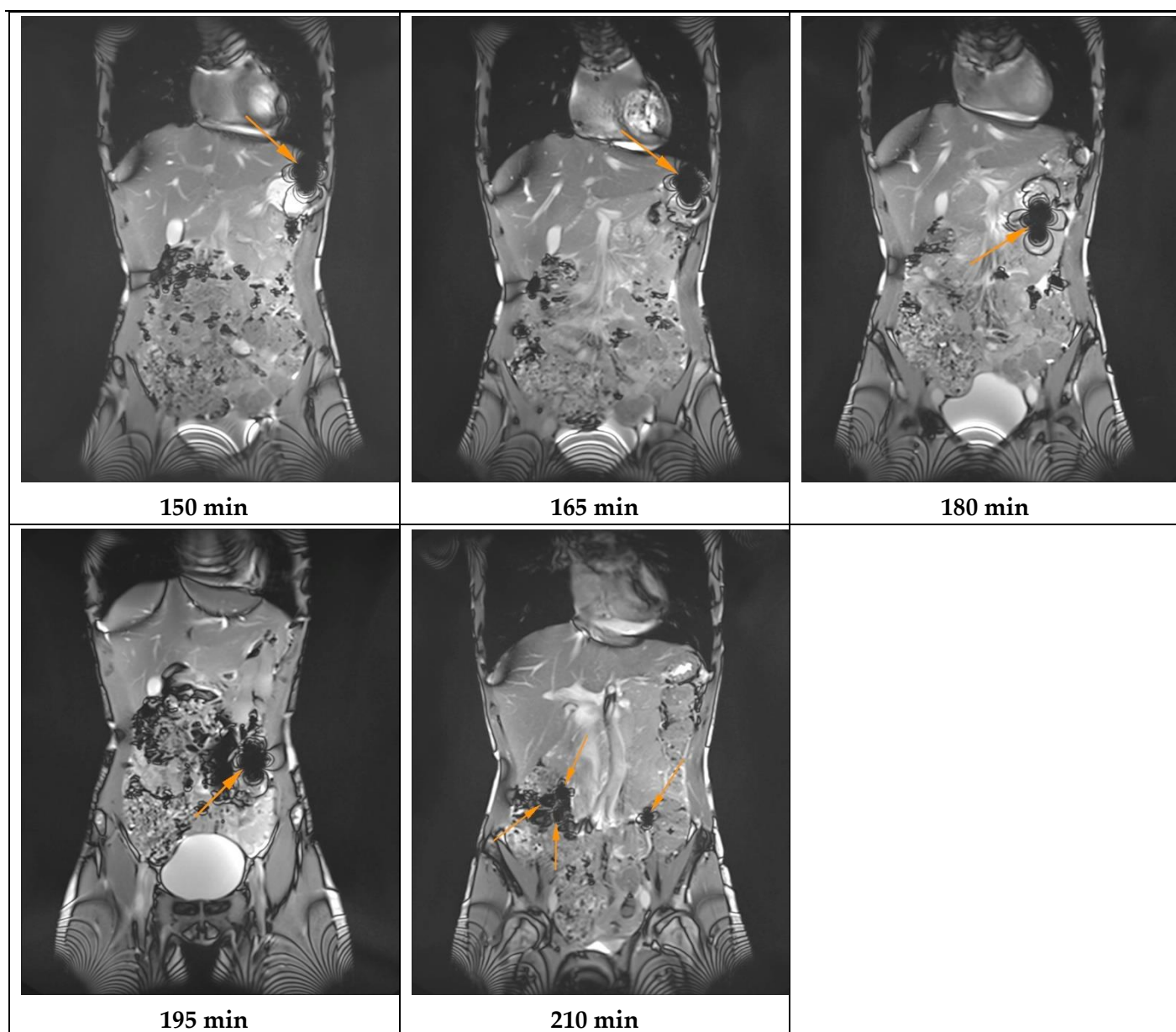

Supplement: Supplementary file 1 [file pharmaceutics-15-02576-s001.zip › pharmaceutics-2602663 - supplementary file7.pdf]

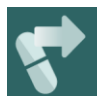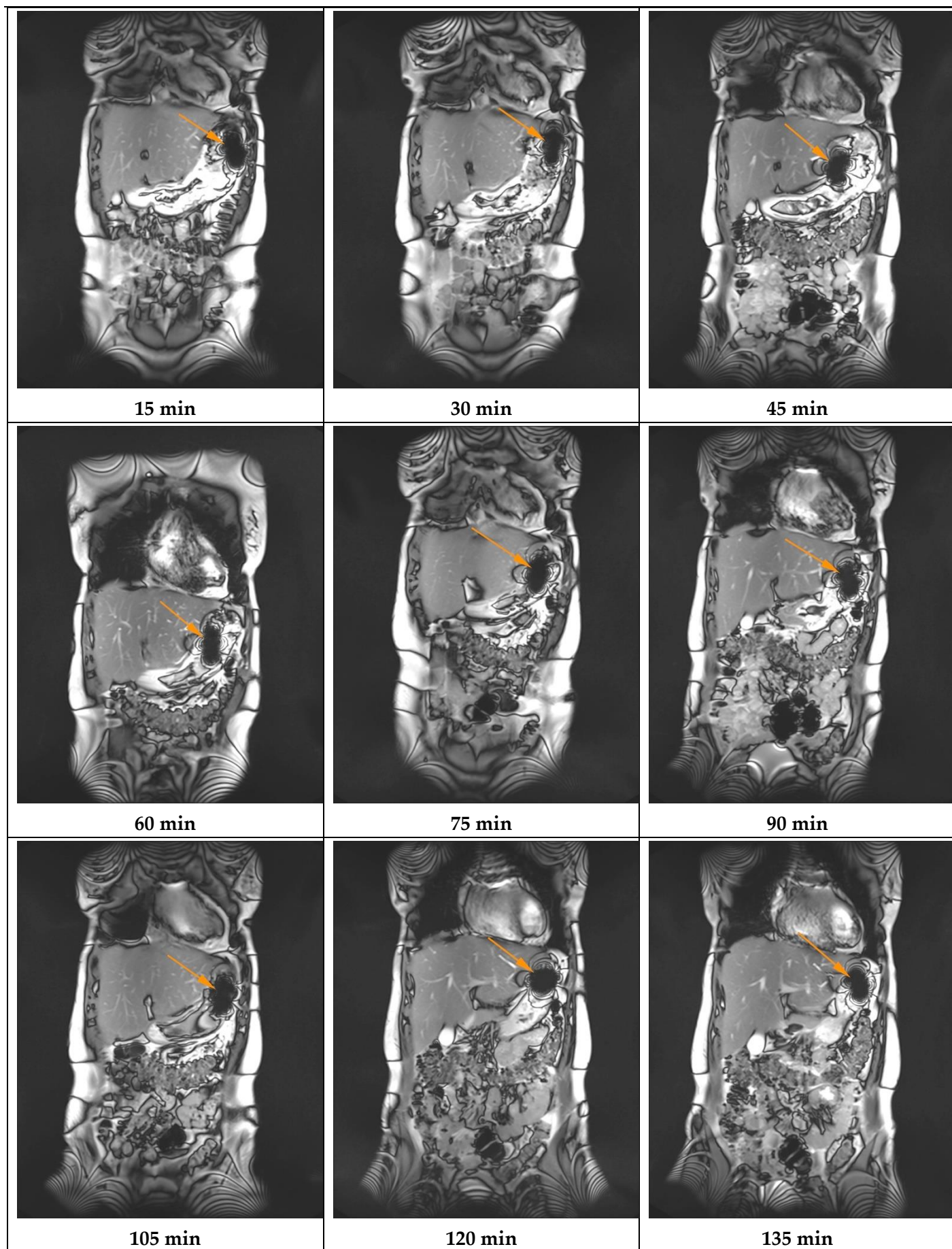

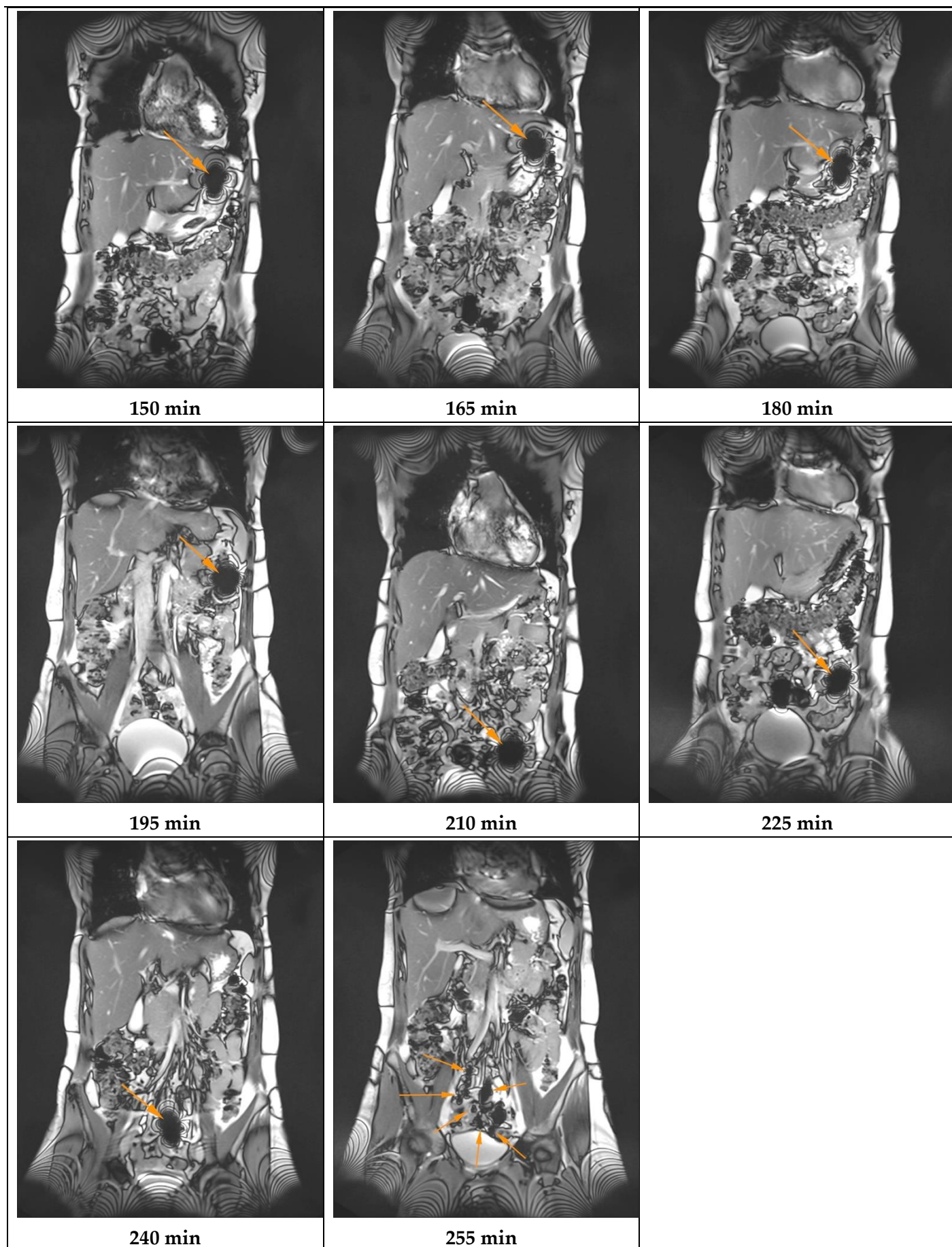

Supplement: Supplementary file 1 [file pharmaceutics-15-02576-s001.zip › pharmaceutics-2602663 - supplementary file8.pdf]

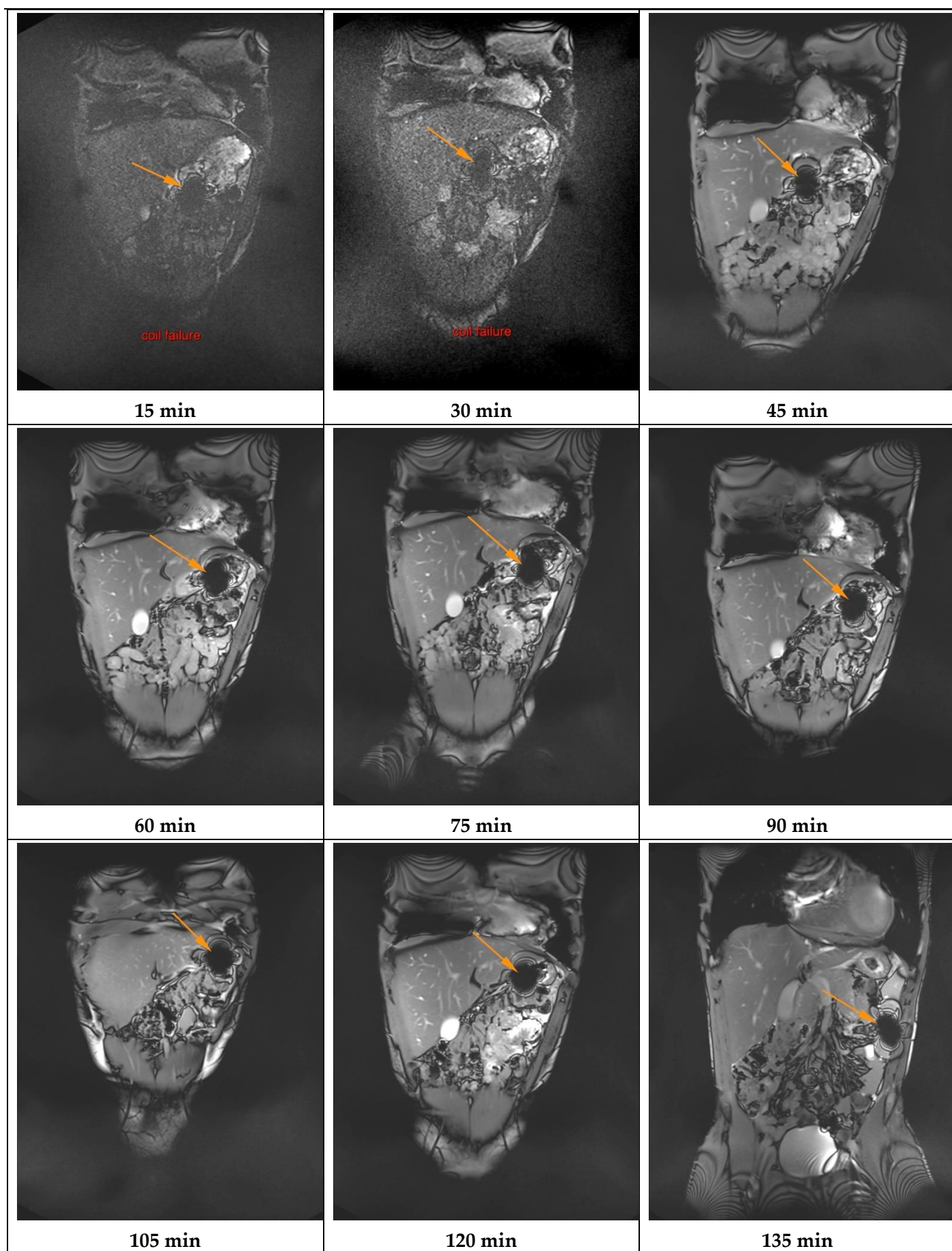

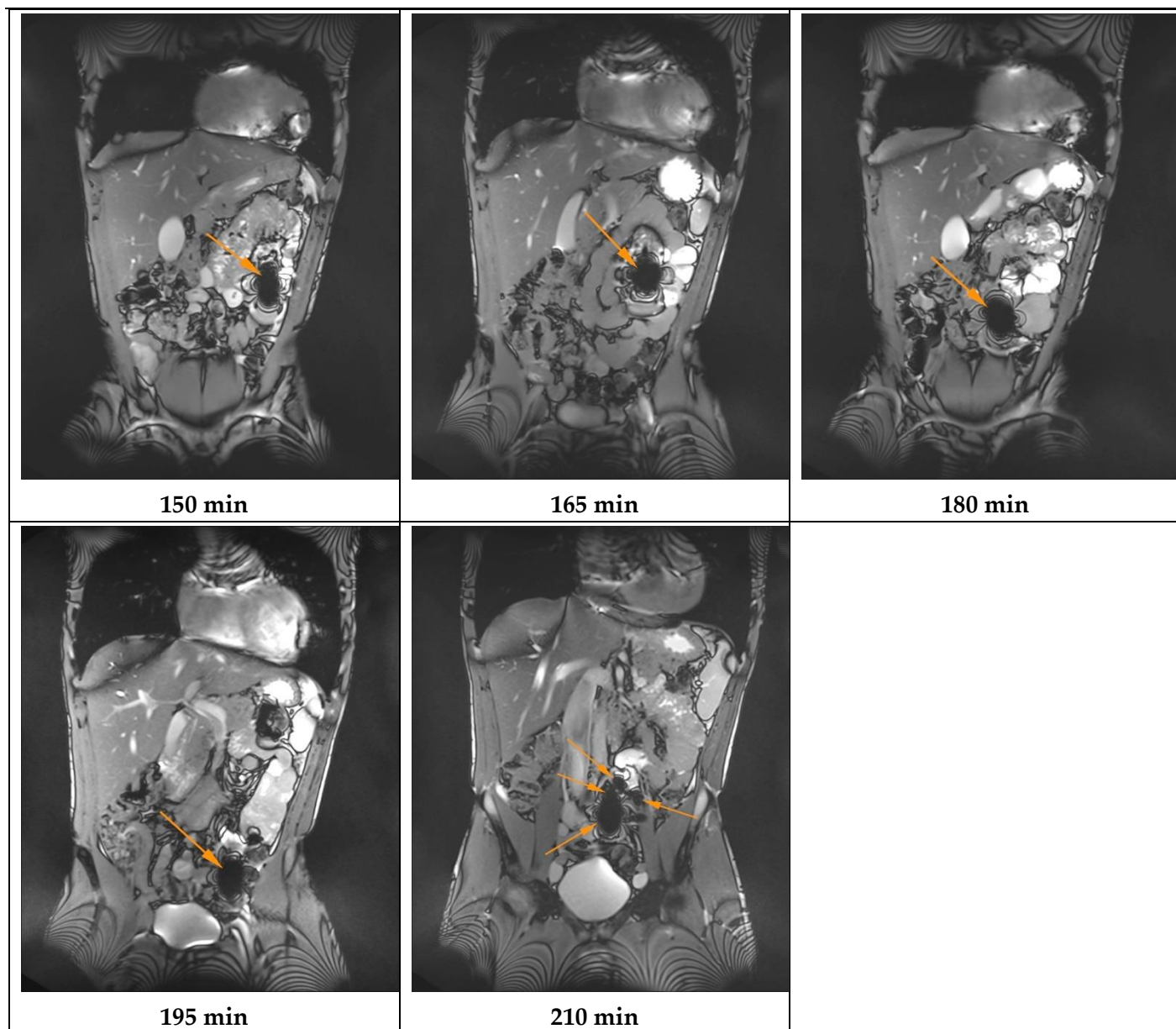

Supplement: Supplementary file 1 [file pharmaceutics-15-02576-s001.zip › pharmaceutics-2602663 - supplementary file9.pdf]
